# Telomere Roles in Fungal Genome Evolution and Adaptation

**DOI:** 10.3389/fgene.2021.676751

**Published:** 2021-08-09

**Authors:** Mostafa Rahnama, Baohua Wang, Jane Dostart, Olga Novikova, Daniel Yackzan, Andrew Yackzan, Haley Bruss, Maray Baker, Haven Jacob, Xiaofei Zhang, April Lamb, Alex Stewart, Melanie Heist, Joey Hoover, Patrick Calie, Li Chen, Jinze Liu, Mark L. Farman

**Affiliations:** ^1^Department of Plant Pathology, University of Kentucky, Lexington, KY, United States; ^2^State Key Laboratory for Ecological Pest Control of Fujian and Taiwan Crops, College of Plant Protection, Fujian Agriculture and Forestry University, Fuzhou, China; ^3^Department of Biological Sciences, Eastern Kentucky University, Richmond, KY, United States; ^4^Department of Computer Sciences, University of Kentucky, Lexington, KY, United States

**Keywords:** *Magnaporthe*, rice blast, wheat blast, subtelomere, telomere instability

## Abstract

Telomeres form the ends of linear chromosomes and usually comprise protein complexes that bind to simple repeated sequence motifs that are added to the 3′ ends of DNA by the telomerase reverse transcriptase (TERT). One of the primary functions attributed to telomeres is to solve the “end-replication problem” which, if left unaddressed, would cause gradual, inexorable attrition of sequences from the chromosome ends and, eventually, loss of viability. Telomere-binding proteins also protect the chromosome from 5′ to 3′ exonuclease action, and disguise the chromosome ends from the double-strand break repair machinery whose illegitimate action potentially generates catastrophic chromosome aberrations. Telomeres are of special interest in the blast fungus, *Pyricularia*, because the adjacent regions are enriched in genes controlling interactions with host plants, and the chromosome ends show enhanced polymorphism and genetic instability. Previously, we showed that telomere instability in some *P. oryzae* strains is caused by novel retrotransposons (MoTeRs) that insert in telomere repeats, generating interstitial telomere sequences that drive frequent, break-induced rearrangements. Here, we sought to gain further insight on telomeric involvement in shaping *Pyricularia* genome architecture by characterizing sequence polymorphisms at chromosome ends, and surrounding internalized MoTeR loci (relics) and interstitial telomere repeats. This provided evidence that telomere dynamics have played historical, and likely ongoing, roles in shaping the *Pyricularia* genome. We further demonstrate that even telomeres lacking MoTeR insertions are poorly preserved, such that the telomere-adjacent sequences exhibit frequent presence/absence polymorphism, as well as exchanges with the genome interior. Using TERT knockout experiments, we characterized chromosomal responses to failed telomere maintenance which suggested that much of the MoTeR relic-/interstitial telomere-associated polymorphism could be driven by compromised telomere function. Finally, we describe three possible examples of a phenomenon known as “Adaptive Telomere Failure,” where spontaneous losses of telomere maintenance drive rapid accumulation of sequence polymorphism with possible adaptive advantages. Together, our data suggest that telomere maintenance is frequently compromised in *Pyricularia* but the chromosome alterations resulting from telomere failure are not as catastrophic as prior research would predict, and may, in fact, be potent drivers of adaptive polymorphism.

## Introduction

The termini of eukaryotic linear chromosomes are protected by “capping” structures termed telomeres, which usually comprise tandem copies of a simple sequence motif - typically (CCCTAA/TTAGGG)_*n*_ although sequence variants are present in many phylogenetic lineages (Peska and Garcia, 2020; Cervenak et al., 2021). New repeats are added to chromosome ends by telomerase - a specialized reverse transcriptase that extends the existing telomere using an RNA subunit that is part of the holoenzyme (Greider and Blackburn, 1989). The primary function of telomeres is to counter the loss of terminal sequence from the lagging DNA strand that occurs naturally during replication - the so-called “end-replication problem.” A second function is to prevent the chromosome ends from being processed as double-strand breaks which could lead to resection by exonucleases, or illegitimate repair by the non-homologous end-joining, or break-induced repair machinery. Protection is accomplished through non-covalent interactions with a number of non-histone, telomere-binding proteins, which together constitute a complex known as “shelterin” (de Lange, 2005).

**FIGURE 1:**
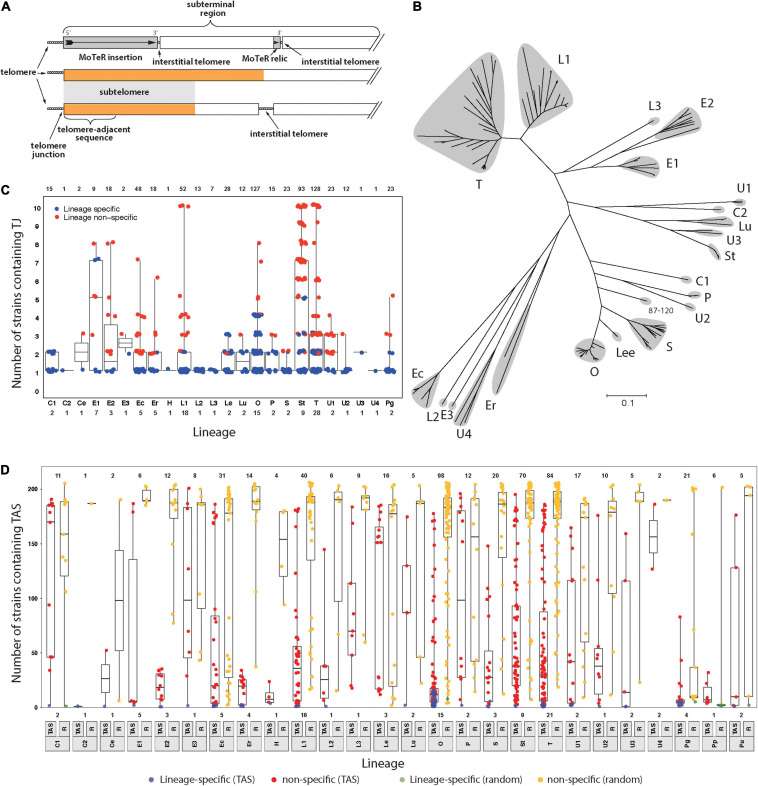
Members of the *Pyricularia* species exhibit poor conservation of telomere junctions and adjacent sequences. **(A)** Schematic explaining telomere-related terminology. Telomeres = sequences at the chromosome ends; telomere-adjacent sequences (TAS) = immediately adjacent, and attached, to telomeres; telomere junctions (TJs) = boundaries between the telomeres and TAS; subterminal regions = portions of chromosomes near to the ends. These can be arbitrary lengths; subtelomeres = sequences present in the subterminal regions and duplicated at multiple chromosome ends; interstitial telomeres = telomeric sequence motifs at internal chromosome positions that can be generated by MoTeR insertion, MoTeR relic formation, telomere internalization, or possibly through random sequence matches. The orange color is used to represent a specific sequence motif, to illustrate how the “subtelomere” designation strictly only extends to subterminal sequences shared with at least one other chromosome end. **(B)** Neighbor joining tree showing genetic relationships between different *P. oryzae* lineages based on whole genome SNP analysis. Branch lengths correspond to pairwise distances between isolates (% nucleotide divergence). Gray “clouds” highlight the major *host-specialized* lineages and are labeled according to most common host (C = *Cynodon*; E = *Eleusine*; Ec = *Echinochloa*; Er = *Eragrostis*; L = *Lolium*; Lee = *Leersia*; Lu = *Luziola*; O = *Oryza*; P = *Panicum*; S = *Setaria*; St = *Stenotaphrum*; T = *Triticum* and U = *Urochloa*). Distinct lineages that came from the same host are given different numerical suffixes. See Supplementary Figure 1 for the expanded *Pyricularia* tree. **(C)** Boxplots showing the distribution of specific TJs among isolates. Each datapoint represents a single TJ identified in a given isolate. Plots are organized according to phylogenetic lineages/species (see labels below *x*-axis). Blue color indicates that the telomere junction was lineage-specific. Box boundaries show the 25th and 75th percentiles and the horizontal line the median value. Note that TJs linked to MoTeR sequences were excluded from this analysis. **(D)** Boxplots showing the distribution of telomere adjacent sequences (TAS) and random sequences among isolates. TAS were filtered to remove repeated sequences and then used to search masked genome sequences. An equivalent number of random sequences (RS) were sampled for comparison. Each datapoint shows the numbers of blast matches found for each TAS/RS identified in a given isolate. Plot organization and percentiles are as in panel **(B)**. Blue and green colors respectively indicate that the TAS or RS were lineage-specific. Values at the top indicate numbers of TAS analyzed. Values at the bottom show numbers of strains analyzed from each lineage/species (lineage labels are shown below the *x*-axis).

The sequences found adjacent to the telomeres are often called “subtelomeres.” Strictly speaking, however, for a sequence to be classified as “subtelomeric,” it should contain motifs that only occur near to chromosome termini, and should be duplicated at multiple ends (Louis, 2014; Figure 1A). True subtelomeres in many organisms have a domain structure, with distal segments containing short, subtelomere-specific, tandem repeat motifs, and proximal segments harboring a number of genes that are duplicated at multiple chromosome ends (Pryde et al., 1997). In many microbes, the proximal subtelomeres contain highly divergent gene families that code for proteins with adaptive benefits – so called contingency genes (Barry et al., 2003). These regions often harbor lineage- or species-specific sequences (Fabre et al., 2004) and can be repositories for genes acquired from related species (Naumova et al., 2005, 2011). Obligate parasites causing human diseases such as malaria and sleeping sickness have large families of highly duplicated genes coding for major surface proteins in their subtelomeres (Leech et al., 1984; Freitas-Junior et al., 2000; Gardner et al., 2002; Donelson, 2003; Berriman et al., 2005). It is believed that duplication and divergence of the resident genes is facilitated by enhanced chromosomal dynamicism in these regions (Pays et al., 1983; Robinson et al., 1999). Additionally, the subtelomeric epigenome environment allows for tight regulation, and occasional switching of expression between different gene copies, which allows these organisms to evade their hosts’ immune system (Freitas-Junior et al., 2005; Figueiredo et al., 2008).

There is emerging evidence that the subtelomere regions play adaptive roles in fungal pathogens. *Pneumocystis jiroveci* (formerly known as *P. carinii*), like its protist cousins, possesses large, highly variable, subtelomeric gene families coding for surface proteins with tightly regulated, yet switchable, expression patterns (Keely et al., 2005). For fungi that are facultative parasites, however, the subtelomeric landscape does not seem to be so heavily invested in the accumulation of specific gene families. Instead, it seems that these regions tend to accumulate certain classes of gene with functional, but not necessarily sequence, similarity - the *Candida albicans* chromosome ends contain a TLO gene family that codes for factors involved in the regulation of virulence (Anderson et al., 2015; Flanagan et al., 2018). Many *Aspergillus fumigatus* chromosome ends contain diverse clusters of genes involved in secondary metabolism (Nierman et al., 2005) which, by their very definition, serve adaptive roles. It appears that this general pattern holds true also for fungal pathogens of plants. Several quantitative trait loci for virulence in the barley pathogen, *Pyrenophora teres f. teres* map in the terminal portions of chromosomes, and are associated with “accessory” genes that exhibit extensive presence/absence polymorphism among isolates (Wyatt et al., 2020).

The fungus *Pyricularia oryzae* (synonymous with *Magnaporthe oryzae*) is a pathogen of cereal crops, turf and weedy grasses and comprises a number of phylogenetically distinct and genetically diverged populations that are mostly host-restricted (Gladieux et al., 2018). Interestingly, approximately 50% of the known genes that determine an isolate’s ability to infect different host genera reside very close to the chromosome ends (Farman, 2007), with the best example being the *Avr-Pita* gene, which prevents infection of rice cultivars possessing the *Pi-ta* resistance gene (Orbach et al., 2000). *Avr-Pita* is positioned just 48 bp away from a telomere. For *P. oryzae*, the telomere proximity of genes that absolutely determine infection capability is of special interest because host specificity is highly fluid and gains of virulence to formerly resistant plants occur with alarming rapidity and frequency (Bonman, 1992). Coupled with this, the chromosome ends are the most highly polymorphic regions of the *P. oryzae* genome, as evidenced by telomere fingerprint variation among closely related strains (Farman and Kim, 2005), as well as frequent presence/absence polymorphism for the sequences found immediately adjacent to the telomeres (Farman et al., 2014). The potential adaptive benefit of this telomere dynamicism is perfectly illustrated by the *AVR-Pita* gene, because spontaneous *avr-pita* mutants with virulence to *Pi-ta* plants were easily recovered, and several had chromosome truncations that evicted *AVR-Pita* (Orbach et al., 2000).

The chromosome ends of some *P. oryzae* strains show such remarkable fluidity that rearrangements of telomeric restriction fragments are readily identified among clonal individuals that have been recently purified by single-spore isolation (Farman et al., 2014; Rahnama et al., 2020). In some experiments, every sibling spore exhibited a telomere fingerprint that was different to the original parent strain (Starnes et al., 2012) and, in the most extreme case, new telomere rearrangements were estimated to occur in 60% of chromosomal molecules (Rahnama et al., 2020). Investigations of this telomere instability led to the discovery of two novel telomere-targeted retrotransposons (MoTeRs) that destabilize the chromosome ends (Starnes et al., 2012). This is primarily because MoTeR insertion generates internal telomere tracts (Figure 1A) that undergo frequent breakage, followed by subsequent rearrangement (Rahnama et al., 2020). Most of the alterations are restricted to the tip-most portions of the chromosomes, however, the discovery of many MoTeR relics at internal chromosome positions (Figure 1A), and their associations with genomic duplications, suggests that MoTeR-mediated telomere destabilization has played a major historical role in shaping the *P. oryzae* genome (Rahnama et al., 2020).

In the present study, we sought to expand on our understanding of how telomere dynamics has impacted *P. oryzae* chromosome architecture and to explore its potential for driving adaptation. First, we took advantage of extensive genome sequence datasets to quantify sequence polymorphism in the immediate telomere-adjacent regions. Second, we leveraged a handful of long-read genome assemblies to gain insight into the chromosomal geography of telomere-related features, and their potential roles in genomic variation. Third, using TERT knockout experiments, we show that many telomere-associated rearrangements can be explained by simple failures in telomere maintenance. Finally, we report the first documented instances of “spontaneous telomere failure,” and provide evidence that these events can provide adaptive benefits.

## Methods

### Sequence Datasets

Sources of genome sequence datasets are listed in Supplementary Table 1.

### Genome Sequencing and Genome Assembly

Genomic DNA was extracted using a standard extraction method (Starnes et al., 2012) and further purified using the Zymo genomic DNA Clean-up kit (Zymo Research, Irvine, CA, United States). Preparation of libraries for Illumina sequencing were performed using the Illumina Nextera or Roche KAPA HyperPlus kits according to the manufacturers’ instructions. Sequencing was performed using MiSeq machines at the University of Kentucky or Bluegrass Community and Technical College; or HiSeq2000 at Novogene (Sacramento, CA, United States). For MinION sequencing, the extracted genomic DNAs were subsequently purified using the MagAttract HMW DNA purification kit (Qiagen, Germantown, MD, United States) and libraries were constructed using the Ligation Sequencing Kit according to the manufacturer’s instructions. Reads were acquired for ∼ 16 h.

Illumina reads were trimmed and residual adaptor sequences were removed using Trimmomatic. Genome assembly was performed using Velvet version 1.2.10 (Zerbino and Birney, 2008) using VelvetOptimiser^1^ to determine the optimal kmer length and coverage. The VelvetOptimiser parameters employed were -s 89 -e 129 -× 2 -shortPaired.

MinION reads were assembled using Canu (Koren et al., 2017) with default parameters. Nucmer and mummerplot (Kurtz et al., 2004) were then used to align the draft assemblies with a chromosome-level reference genome for strain LpKY97, which allowed the contigs to be re-ordered in co-linear fashion. The resulting chromosome-level draft assemblies were then interrogated to identify rare translocations that caused telomeres to align with “internal” positions on the reference genome. If an internally aligned telomere defined the end of a contig in the draft chromosome, the assemblies was “broken” at this position, so that the telomere correctly defined the new chromosome end. To identify breaks at interstitial telomeres, MinION reads were aligned with the US71 reference genome using Minimap2 (Li, 2018) and visualized in IGV (Robinson et al., 2011).

### Phylogenetic Analysis

Assembled genomes were masked using a custom masking algorithm from the TruMatch blast post-processor (Li et al., 2005). Masked genomes were then aligned in pairwise fashion and SNPs were called using the iSNPcaller program^2^. The resulting normalized pairwise distance data (SNPs per megabase of uniquely aligned sequence) were used to build a neighbor joining tree using MEGA X software (Kumar et al., 2018). The phylogenetic lineages thus identified were named according to the majority host from which the constituent members were isolated, with numerical suffixes being used to distinguish different lineages from the same host.

### Identification of Telomere Consensus Sequences

Telomere-containing sequence reads were retrieved from raw, paired-end, FASTQ format Illumina datasets using a simple grep search to retrieve reads with exact matches to CCCTAA_3_ and TTAGGG_3_. Reads containing the latter motif were reverse complemented before clustering the reads using wcdest (Hazelhurst et al., 2008). The resulting clusters were then assembled with MUSCLE (Edgar, 2004) using default parameters, and consensus sequences (telConsensus) were called using a custom script. Nucleotide positions not reaching a majority consensus were represented with Ns. Internal telomere-like sequences were identified as telConsensus that did not start with any permutation of the CCCTAA motif and were discarded from further analysis. Information about the raw read datasets and the strains from which they were derived are provided in Supplementary Table 1.

### Characterization of *de novo* Telomeres

Raw reads that started with telomere repeats but did not cluster with other telomeric reads, or belonged to very small clusters were considered candidates for *de novo* telomeres that formed while the strain was being cultured for DNA extraction. These reads were searched against the telConsensus using the low stringency default blast parameters to filter out reads that escaped clustering due to poor quality sequence. The resulting reads were then searched against the relevant genome to identify their original chromosomal positions.

### Surveying for Telomere Junctions in Raw Illumina Sequence Datasets

To detect the presence of individual telomere junction (TJ) sequences (Figure 1A) in different *Pyricularia* strains, we used SeqOthello - a program that accelerates searching of very large raw (FASTQ) sequence datasets using a fast and efficient indexing structure (Yu et al., 2018). Using telConsensus as inputs, “SeqOthello,” iterates through all possible 21-mer subsequences and tests for their presence in raw read datasets, thereby eliminating issues associated with poor telomere assembly. Here it is important to note that single nucleotide polymorphisms in a given SeqOthello search window can prevent detection of TJ conservation. However, for this to be a problem in the present study, a SNP would have to occur within 21 nucleotides of the junction (the query k-mer length used by SeqOthello). This is unlikely when most *Pyricularia* isolates within a species show much less than 1% sequence divergence, and such instances would be detectable in the outputs. Telomeres attached to MoTeR sequences were removed from the analysis because repeated MoTeR insertion events can give false impressions of TJ preservation. Annotated data outputs are summarized in Supplementary Datasheets 1, 2.

### Surveying for Telomere-Adjacent Sequences in Assembled *Pyricularia* Genomes

Telomere repeats were stripped off the start of the telConsensus sequences to generate a set of telomere-adjacent sequences (TAS, Figure 1A) which were then used as queries in BLAST (RRID:SCR_001598) searches of more than 200 *Pyricularia* strains. Information about these strains and access to the sequence data is provided in Supplementary Table 1. To avoid overcalling matches caused by repeated sequences which are often enriched in subterminal regions, we used genomes where all sequences with a copy number > 1 were masked. Valid hits were scored as alignments that covered ≥ 90% of the query. For comparison, a dataset comprising equivalent numbers of randomly selected 280 bp sequences were retrieved from each of the repeat-masked test genomes. Sequences containing NNNs (indicative of gaps/repeats) were discarded and resampled. The random sequence queries were searched against the subject genomes using parameters that were identical to the TJs. As before, valid hits were scored as alignments that covered ≥ 90% of the query. The BLAST results that matched the filtering criteria are provided in Supplementary Datasheet 3.

### Genomic Migrations of Telomere-Adjacent Sequences

Telomere-adjacent sequences were used as queries in BLAST searches of the CD156 MinION reference genome (NCBI: BioProject PRJNA320483). Sequences with multiple matches to the genome were filtered out, as were redundant TAS, and the positions of non-redundant sequences having unique “hits” were plotted along the chromosomes using a custom R script^3^. Vertical displacement of overlapping datapoints was performed using the ggplot “jitter” function to resolve hits with similar positions. The unique BLAST hits used for plotting are provided in Supplementary Datasheet 4.

### Identification of MoTeR Relics

BLAST searches (-*e*-value 1e-1 -task BLASTn-short) were used to reveal the locations, lengths, and orientations of MoTeR relics in genomes. Matching sequences were considered relics if they did not belong to telomeric MoTeRs arrays. UNIX grep searches of the MoTeR 3′ end was used to detect matches that were missed by BLAST due to short match lengths. The positions of MoTeR relic-associated duplications are provided in Supplementary Table 2.

### Analysis of Duplications Flanking MoTeR Relics

BLASTn (*e*-value = 1e-20) interrogations of the CD156 genome against itself were used to search for duplicate sequences. Duplications linked to MoTeR relics were detected using the Integrative Genomics Viewer (Thorvaldsdottir et al., 2013). Duplications were considered MoTeR-associated if the secondary alignment started within 20 nt of a relic boundary and were retained for further analysis if they were at least 500 bp in length. MoTeR relics and adjacent duplicate flanking sequences were visualized using the R package Circos (RRID:SCR_011798) (Krzywinski et al., 2009). The positions of MoTeR relic-associated duplications are provided in Supplementary Table 3.

### Chromosomal Distributions of Telomere Relics

Candidates for former telomeres that had been internalized (telomere relics) were identified using a python script to search MinION assemblies for all permutations of the repeat motif (CCCTAA/TTAGGG, CCTAAC/GTTAGG, … etc.) that contained at least 2, 3, or 4 repeat units see text footnote 3. An inexact matching algorithm was employed to account for occasional sequence errors and matches to telomeres and interstitial telomeres in MoTeR arrays were filtered out. The positions of matches on the respective chromosomes were then scaled to allow their relative distances to the telomere to be plotted on a single reference assembly. The relative positions of telomere relics are provided in Supplementary Datasheet 5.

### Deletion of the *P. oryzae* Telomerase Gene

A gene disruption construct was created by sandwiching a hygromycin B resistance cassette between 1.5 kb sequences that flanked the telomerase gene (MGG_01617). Briefly the flanking sequences were amplified from genomic DNA, using the following primers, TERT1-F: 5′ GTTCTCTTCCCGCATTTCAG 3′; TERT1-R: 5′ GGCAAGCTTGGAAAGAACTGCT 3′; TERT2-F: 5′ ATTGAGGATCCCGCATTTCAGTCACG 3′; and TERT2-R: 5′ CCATCTCTAGAGCGAGGCTA 3′ and then purified using a PCR cleanup kit (Invitrogen). The 5′ flank was digested with *Hin*d*III* and the 3′ flanking fragment with *Bam*HI. The fragments were gel-purified (Qiagen, Inc.) and then ligated with the hygromycin B resistance cassette that had been released from the backbone vector with *Hin*dIII and *Bam*HI, using a reaction volume of 5 μl. After overnight incubation, the ligation mix was diluted 100-fold and then the full-length disruption cassette was amplified using the TERT1-F and TERT2-R primer pair. The desired fragment was gel-purified and used directly for transformation.

### Fungal Transformation

Protoplasts were transformed using previously reported methods (Peyyala and Farman, 2006). The hygromycin selection plates were surveyed daily to identify transformants (colonies with smooth, hazy borders), and colonies were carefully monitored for sudden cessation of growth (indicative of telomere crisis). Hyphal tips were then carefully excised, being extremely careful not to include hyphae from neighboring, untransformed individuals. A number of normally growing, “ectopic” transformants were picked as controls.

### Fungal Cultures

Routine fungal growth was performed using oatmeal agar. Single spore isolation was performed by massaging a colony with a sterilized, sealed glass pipette and then streaking across the surface of water agar. After overnight incubation, a single, germinated spore was isolated under the dissecting microscope and transferred to fresh oatmeal agar plate. For DNA extraction, fungal cultures were grown with shaking for 5 to 7 days in test tubes containing 10 ml of liquid complete medium (6 g casamino acids, 6 g yeast extract, 10 g sucrose per liter).

### DNA Extraction and Southern Hybridization

DNA was extracted as previously described (Rahnama et al., 2020). For Southern hybridization, electrophoretically fractionated DNAs were electroblotted to Pall Biodyne B membranes (Pall Corp., Port Washington, NY, United States) using the manufacturer’s instructions (Idea Scientific, Minneapolis MN, United States). α^32^P dCTP- labeled probes were prepared by oligolabeling (Thermo Fisher Scientific, Waltham, MA, United States) according to the manufacturer’s instructions and hybridization was performed using previously described conditions (Starnes et al., 2012).

### Plant Growth and Inoculation

Seeds of rice cultivar 51583 were soaked in de-ionized (DI) water overnight, sterilized for 10 min by shaking in a 50% bleach solution, and then rinsed in DI water. Pots (2.35″ × 2.15″ × 2.33″) were filled with moistened coarse ground vermiculite and 12-15 seeds were sowed in each pot. The pots were labeled and placed in plastic trays flooded with DI water and covered with a transparent plastic lid. The trays were incubated in a growth chamber using a 27°C, 16 h/21°C, 8 h light (400-500 μE/m-2s-1)/dark cycle with humidity at < 80%. After seedling emergence (∼7 days), the plastic cover was removed, and the trays were watered daily with Hoagland’s solution. Plants were inoculated when the third leaf emerged (approximately 2 weeks post-planting).

Fungal cultures were activated from frozen stocks by placing paper disks on oatmeal agar. Cultures were then grown at 25°C under continuous illumination. After 14 days, the plates were flooded with 10 ml of 0.25% gelatin, the surface of the colony was massaged with a sterilized cell spreader to liberate conidia and the solution was filtered through Miracloth (EMD Millipore, San Diego, CA, United States). Spores were quantified using a hemocytometer and adjusted to 10^5^/ml with 0.25% gelatin.

Plants were placed in a single Mycobag (Garland, TX, United States) containing a few milliliters of water to increase humidity. Aerosol inoculation was conducted using a glass sprayer at 20 psi. The bags were sealed and incubated in the dark for 20 h at 22°C. The bags were then moved to the growth chamber and opened slightly to equilibrate humidity. After 1 h, the pots were removed and maintained in the growth chamber as above. Disease observations and ratings were made 7 days post-inoculation.

## Results

### Extreme Variability in *Pyricularia* Chromosome Tip Structure Suggests That the Chromosomes Experience Occasional “Telomere Failure”

A previous analysis of *P. oryzae* chromosome ends performed in the early days of genome sequencing revealed that the sequences found immediately adjacent to the telomeres (telomere-adjacent sequences, or TASs, Figure 1A) in one strain were often absent from other strains of the fungus; or, if they were present, they were not located at telomeres (Farman et al., 2014). On the other hand, the fact that each strain had “unique” TAS indicated that the chromosome ends are regions of enhanced genomic innovation. Here, we wished to explore the full extent of this telomeric variability by examining genome sequence data from a comprehensive collection of *Pyricularia* strains/species (*P. oryzae*, 138 isolates; *P. grisea*, 4 isolates; *P. pennisetigena*, 2 isolates; and *P. urashimae*, 2 isolates). Altogether, these represent 25 phylogenetic lineages (Figure 1B and Supplementary Figure 1) and are variously specialized on 17 different host plant genera (Supplementary Table 1).

First, we focused on the very tips of the chromosomes, with the goal of determining how often different strains have precisely the same telomere structure at a given chromosome end. To this end, we first performed targeted assemblies of telomeric sequences for each strain. Then for each resulting contig, we used SeqOthello (Yu et al., 2018) to ask if the sequence that defines the junction between the telomere and the telomere-adjacent sequences (TAS) is present in any of the other isolates. Among 689 TJs analyzed, we found that 331 (48%) were “private” - i.e., they occurred only in the strain of original discovery. Another 140 TJs showed lineage-specific conservation and, even when junctions were shared, they were not widely distributed because the median number of strains possessing a given TJ was just 3.2 (Figure 1C).

### Telomere-Adjacent Sequences Are Poorly Conserved Among Different *Pyricularia* Strains

One possible explanation for the poor conservation of TJs is that the *P. oryzae* chromosomes experience occasional telomere failure. This, in turn, would make them prone to terminal truncations which would result in a corresponding loss of the telomere-adjacent sequences (TASs). To test for such occurrences, we took the TASs identified in each strain as queries, and used BLAST to test for their presence in 204 *Pyricularia* genome assemblies. As a control, we performed a parallel analysis with equivalent sets of sequences sampled randomly from each of the respective genomes. The randomly selected sequences showed high conservation with a median presence in 186 strains (91%). Only seven of the 516 random sequences analyzed showed lineage−specificity (Figure 1D). In comparison, the TASs were very poorly conserved because, among the 516 single-copy TASs analyzed, 38 were private, 471 were lineage-specific, and the median number of strains that possessed a given TAS was only 22 (Figure 1D). Strains from certain hosts had TASs that were much more poorly preserved than others, with those from rice (*Oryza sativa*, O) being prime examples. The median number of strains sharing an O-strain TAS was found only 15, and 39% of the TASs (39/98) showed lineage specificity. TASs found in *P. grisea*, *P. pennisetigena* and *P. urashimae* also showed extremely poor conservation in other species but this was to be expected given the significant sequence divergence between species (∼ 10%), which typically allows only ∼60% of their genomes to be aligned. That most of the TASs identified in this study were absent from a majority of strains is certainly consistent with a pattern of occasional telomere failure.

### Telomere-Adjacent Sequence Mapping on a Reference Genome Reveals Histories of Chromosome Truncations and Duplications of Internal Sequences at Chromosome Ends

**FIGURE 2:**
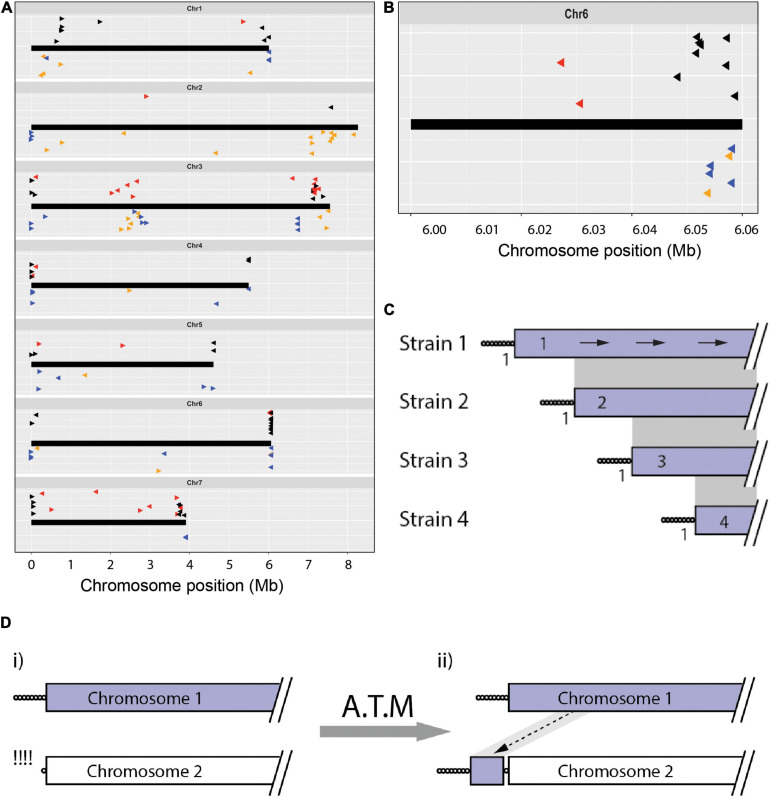
The chromosomal geography of TAS and interstitial telomeres. **(A)** Plots showing the “normal” chromosomal locations of sequences that were found in telomere-adjacent positions in one or more isolates. Chromosomes are represented as thick black lines. Data points plotted on top show the positions on the CD156 chromosomes of sequences occupying telomere-adjacent positions in the *Pyricularia* strains used in TJ analysis (The arrowheads point 5′ to 3′ on the telomeric query sequence - the clipped off telomere would have been at the 5′ end of the arrow). Black arrowheads represent TAS that are single-copy in the source genome, red are duplicated TAS. The dataset was filtered to remove redundant matches where two strains shared identical TJs. Data points on the bottom (Δ) show the relative positions of all interstitial telomere sequences found in the MinION assemblies of Arcadia2, CD156, FH, FR13, Guy11, LpKY97, U233 and US71. Tri-repeats (CCCTAA/TTAGGG)_3+, < 4_ are shown in orange and tetramers (CCCTAA/TTAGGG) _≥ 4_ are shown in blue. Arrowheads pointing right represent CCCTAA repeats and left, TTAGGG. Vertical “jittering” was used to improve the resolution between matches at similar positions. **(B)** TAS alignments on the right-hand end of chromosome 6 showing an organization consistent with progressive chromosome truncations. **(C)** Schematic showing how truncations differentially affect the preservation of TJs and TAS. Depicted are alignments of a single chromosome end that is variably truncated in four strains. Telomere repeats are represented as circles and gray shading connects subterminal sequences that align. Numbers adjacent to the TJs indicate the number of strains that share each junction; and values in the TAS show the numbers of strains containing that TAS. The arrows on the top chromosome show how blast searches with TAS from strains 2, 3, and 4 TAS are expected to produce alignments with identical orientations. **(D)** Schematic showing copying of internal chromosome sequence onto a failed telomere. (i) Chromosome 2 experienced failure and resection of the telomere; (ii) the free end was then repaired via an ATM process that resulted in the copying of internal sequence from chromosome 1 onto the free end.

While 331 TJs were private to the strain in which they were originally identified, only 38 TASs showed this pattern. This naturally begs the question, if a TAS is present in a given genome but is not linked to a telomere, where is it? This was addressed by using the TASs as queries to search a chromosome-level genome assembly for the *Eleusine* pathogen, CD156. The resulting alignments are plotted above the chromosomes shown in Figure 2A. To simplify interpretation of the results, only unique matches were plotted, and the TAS from CD156 were excluded. Eighty-three of the 516 TAS had unique matches in the CD156 genome and most aligned with sequences found in subterminal locations. Predictably, none of the alignments abutted a telomere. In many cases, the positions and orientations of TASs in the CD156 chromosomes were consistent with their having arisen through simple terminal truncations that extended for variable distances in the chromosomes of the source strains (see Figures 2B,C). On the other hand, a number of TASs aligned with positions deep within the CD156 genome interior, or they were in inverted orientation with respect to a nearby telomere. As such, they were not explicable by terminal truncation. In most cases, however, these particular TASs had a second copy in the source genome (Figure 2A), which suggests that these sequences very likely arrived in telomeric positions after having been captured from internal loci during the repair of failed telomeres (Figure 2D; and see below).

Also notable were two situations where TASs from different strains mapped in clusters positioned firmly within the CD156 genome interior. Four strains had TASs that all aligned in the “sense” orientation at distinct positions centered around ∼775 kb from TEL1. Because these sequences were all single-copy in the source genomes, it is clear that chromosome 1 terminated at the corresponding positions in the respective strains. This suggests that they had a common ancestor that experienced a chromosomal break that was healed by *de novo* telomere formation. The new telomere then appears to have suffered variable length truncations (as illustrated in Figure 2C). A second hotspot for telomere-driven alterations lies in a region between 2 and 3 Mb on chromosome 3. Not only do several TASs map there but, CD156 also harbors a number of MoTeR relics and interstitial telomere sequences in this location (see below). The presence of so many “telomeric features” at an internal location, suggests that these might be footprints of an ancestral chromosome fusion event. Note that we were able to rule out mis-assembly of the CD156 genome as an alternative explanation because it was validated via alignment with chromosome-level assemblies for three other strains (B71, LpKY97-1, and FH).

### Overabundances of Internal Telomere Repeat Motifs Suggest That *P. oryzae* Experiences Episodic “Telomere Internalization”

The distributions of other strains’ TASs on the CD156 reference genome pointed to rearrangements driven by the healing and/or repair of chromosome ends that experienced temporary telomere failure. When telomere maintenance fails, replicative sequence attrition results in the progressive loss of terminal sequence until the chromosome ends are healed by *de novo* telomere formation, or via alternative telomere maintenance (ATM) processes that add internal sequences onto de-protected ends. If ATM is activated before the telomere repeats are completely lost, the vestigial telomeres will be internalized (see below). In *P. oryzae*, the canonical telomeres have lengths ranging from ∼20 to 30 repeats (Rehmeyer et al., 2006; Starnes et al., 2012) but, for internalized (interstitial) telomeres, we would expect their lengths to be shorter because repair is not activated until they reach a critical length (∼10 repeats). To identify possible telomere immigrations, we searched chromosomal assemblies of eight strains for interstitial telomere motifs with the various permutations of at least two, three and four repeat units (CCCTAACCCTAA, CCTAACCCTAAC, etc.). On average, bi-repeats are expected to have a random occurrence of approximately six times every 16.8 million nucleotides, or ∼ 36 times in a ∼ 37 - 45 Mb double-stranded genome. Actual occurrences far exceeded random expectations, with an average of 119 per genome (range 73 to 265, Table 1). Tri-repeats and larger also occurred much more frequently than by random expectation (1 in 68.7 Gb), with an average of 10 discrete copies per genome (range 2 to 21, Table 1). These overabundances suggest that a number of these motifs represent former telomeres that were internalized when they were repaired following deprotection. Plotting the relative positions and orientations of these motifs against the CD156 reference genome provided additional support for this hypothesis because it showed that the most of the interstitial sequences were in subterminal regions; and a majority of these were in the orientation expected had they been former telomeres that were repaired after experiencing failure (Figure 2A below chromosome and Figure 2B).

**TABLE 1:** Telomere motifs in the genome interior.

Genome assembly	(CCCTAA/TTAGGG)_2_	(CCCTAA/TTAGGG)_3+_	Genome size (bp)
Arcadia2_Final	88	9	43,775,141
CD156_Final	99	9	41,851,933
FHSS2-1_Final_V2	105^*A*^	14	43,716,171
FR13_Final	89	10	43,611,129
Guy11_Final	82	2	42,954,272
LpKY_Full_v4.3	265^*A*^	12	45,266,885
U233_Final	73	5	39,733,298
US71_UCNY03_Final	103	21	43,088,661

*^*A*^Includes minichromosomes.*

### Internalized MoTeR Relics Are Associated With Genome Rearrangements

**FIGURE 3:**
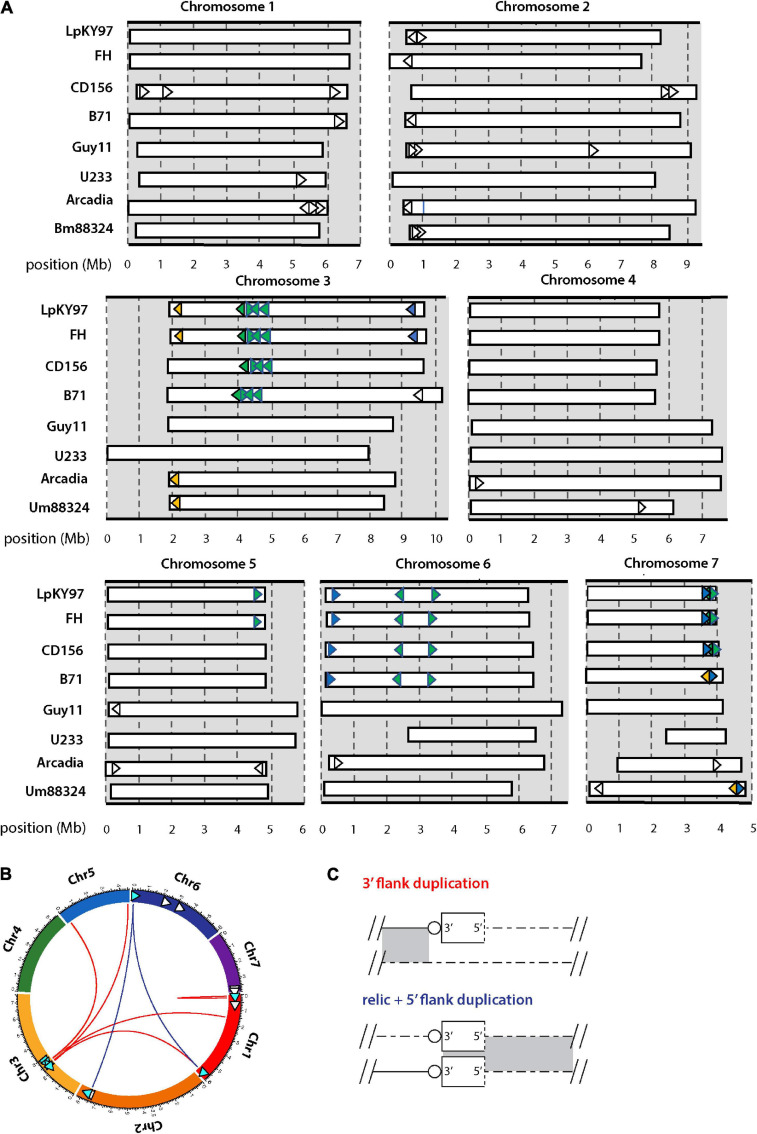
Chromosomal distributions of MoTeR relics and associated duplications. **(A)** Chromosomal locations of MoTeR relics. Note: telomeric MoTeRs are not shown. Shared insertions are shown in the same color. Relics with no fill color are unique to the genome in which they are shown. **(B)** Circos plot showing the secondary positions of sequence duplications flanking MoTeR relics. Relics are shown with arrowheads pointing 5′ to 3′. Those with associated duplication have a blue fill color. Colored lines connect the relic to the location of the duplicated sequence(s), with line color denotes the type of duplication, as shown in panel **(C)**. **(C)** Duplications surrounding a MoTeR2 relic locus are classified according to whether a duplication extends into the MoTeR 5′ or 3′ flank, or both. Shown are alignments between a MoTeR relic locus top and a secondary locus elsewhere in the genome. Relics are shown as rectangles with the dotted line at the 5′ terminus representing the truncated MoTeR border. Telomere repeats at MoTeR 3′ termini are shown as circles. Matching sequences are connected by gray shading.

While overabundances of internal (CCCTAA)_*n*_ motifs are certainly consistent with the internalization of former telomeres, we cannot rule out the possibility that these motifs evolved independently and over-accumulated for functional reasons. However, additional evidence in support of telomere internalization comes from the presence of MoTeR relics (Figure 1A) at internal chromosome positions (Rahnama et al., 2020). MoTeRs insert specifically into telomeres (Figure 1A), so the presence of very short, 5′-truncated relics with associated telomere motifs at internal locations is best explained by their having origins in terminal positions (Starnes et al., 2012). CD156, has MoTeR insertions in most of its telomeres and 14 internal relics were found distributed among five of the seven chromosomes (Figure 3A). Approximately half were positioned in subterminal regions (within ∼500 kb from a chromosome end), while six were deeply embedded in the chromosomes, at least 2 Mb from the nearest telomere. Most of the relics in CD156 were associated with segmental duplications and, interestingly, mapping of the secondary copies revealed that most reside in subterminal regions and usually very close to the chromosome ends (Figures 3B,C).

MoTeRs cause telomeres to rearrange at spectacular frequencies (Rahnama et al., 2020), which suggests that relics might be continually generated by “illegitimate” repair. In this case, we might expect different *P. oryzae* strains to be highly polymorphic around relic loci. Using whole genome comparisons, we found that most strains did indeed vary widely in their relic constituency (Figure 3A). Strains B71, FH, and LpKY97, are somewhat exceptional because they share many relic loci with CD156 owing to the fact that they recently inherited significant portions of their genomes from a related *Eleusine* pathogen (unpublished data). Arcadia2 had 11 relics distributed among all chromosomes (Figure 3A), which was surprising because it doesn’t have any functional MoTeRs in its telomeres (just one drastically truncated insertion in TEL10). All 11 copies resided close to chromosome ends but none matched copies found in CD156. US71 - a strain related to Arcadia2 - also contained 11 relics but these were distributed across five chromosomes, and only five matched copies in Arcadia2. The rice pathogen, Guy11, had just four relics, while U233, from St. Augustinegrass - had only one (Figure 3A).

If the wide strain-to-strain variation in relic copy numbers and positions were due to independent internalization events, we would predict that polymorphic relics define duplication or translocation breakpoints detectable using genome alignments. However, a comparison of CD156 (14 relics) with U233 (1 copy) revealed that, in every case, U233 failed to possess the subterminal relics because the chromosome ends comprised strain/lineage-specific sequences. On the other hand, the more “internal” relics were usually absent due to deletions in the U233 genome where chromosomal synteny was preserved across the loci, but the relics and varying amounts of flanking sequence were simply absent (Table 2). The same pattern held true for most other instances where a relic was found in one genome but not another (Table 2). In a few cases, relic deletion was associated with a larger rearrangement such as a translocation or an inversion, which possibly could have been coincident with, and driven by, MoTeR internalization, although the absence of the relic precluded a definitive conclusion in this regard.

**TABLE 2:** Basis for strain-to-strain polymorphism at MoTeR relic loci present in the CD156 genome.

Chr#	3′ end position	Relic length	Sequence of relic 3′ end + telomere vestige	Disposition in other strains^*A*^		
				Arcadia2	US71	U233
1	215205	40	CGCGAATTAAAACCCTAACCCTTA	TSS	TSS	TSS
1	5749080	40	CGCGAATTAAAA**CCCTA**TA^*E*^	ID	ID	ID
2	7550702	31	CGCGAATTAAAA**CCCTAAC**	TL+D	TL+D	TL+D
2	7553167	40	CGCGAATTAAAA**CCCTA**	TL+D	TL+D	TL+D
3	2424482	40	**AGGGTTAGGG**TTTTAATTCGCG	ID^*B*^	TL+D^*C*^	ID^*B*^
3	2621949	40	CGCGAATTAAAA**CCCTAACCCTA**	ID^*B*^	TL+D^*C*^	ID^*B*^
3	2625549	41	**GTTAGGG**TTTTAATTCGCG	ID^*B*^	TL+D^*C*^	ID^*B*^
3	2660716	31	**GGTTGGGG**TTTTAATTCGCG	ID^*B*^	TL+D^*C*^	ID^*B*^
3	7124927	15	**TTAGGG**TTTAATTCGCT	TL+D	TL+D	TSS
6	95554	31	CGCGAATTAAAA**CCCTAA**	TSS	TSS	TL+TT
6	2153119	40	**TTGGGG**TTTTAATTCGCG	ID	ID	TL+ID
6	3282214	132	CGCGAATTAAAA**CCCTAACC**	ID	ID	ID
7	3761013	38	CGCGAATTAAAA**CCTTAACCCTAA**	INV+D	TL+D	Chr4^*D*^
7	3893775	63	**TTAGGGTGTGGG**TTTTAAATCGCG	TSS	TSS	TL+SS
7	3896604	105	CGCGAATTGAAA**CCCTAACCCTAA**	TSS	TSS	TL+SS

*^*A*^D, deletion (combined with another rearrangement); ID, internal deletion; INV, inversion; SS, sequence substitution; TL, translocation; TT, terminal truncation; TSS, terminal sequence substitution.*

*^*B,C*^All deleted in single rearrangement event.*

*^*D*^Present on chromosome 4.*

*^*E*^Motifs in boldface text are the vestigial telomeres at the 3’ ends of the MoTeR relics.*

**FIGURE 4:**
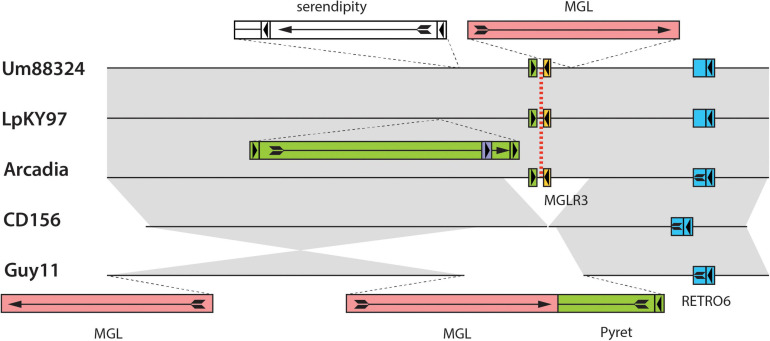
Chromosomal rearrangements at a MoTeR relic locus. Shown are alignments surrounding a MoTeR relic locus at position on LpKY97 chromosome 3. Strain names are listed on the left. The position of the relic is indicated with a red dotted line. Transposon insertions are shown as colored rectangles and their orientations are indicated with arrows. At least one copy of each element is named. Full-length long terminal repeat (LTR) retrotransposons are represented as colored boxes with a complete arrow (head + tail) representing the gag-pol open reading frame (ORF arrow). Smaller, flanking boxes with arrowheads represent the LTRs. Solo-LTRs are represented as appropriately colored small boxes with directional arrowheads. Truncated elements are shown missing LTRs and truncated ORF arrows. Full-length copies of the LINE-type MGL element are represented as plain red rectangles with a complete ORF arrow showing the reverse transcriptase open reading frame. Gray shading is used to connect alignable sequences.

A nice example of relic-related polymorphism is shown in Figure 4. This shows a relic on chromosome 3 of the *Lolium* pathogen LpKY97 that is conserved in Arcadia2 and Um88324 (signalgrass) but absent in CD156 and Guy11. Alignments of the five loci revealed that overall chromosomal synteny was preserved in all cases but the relic was simply deleted in CD156 and Guy11 (Figure 4). In Guy11, the deletion breakpoints were defined by insertions of the MGL and Pyret retroelements, whereas in CD156, the breakpoints occurred in a nondescript, single-copy sequence. This suggested that the deletion in CD156 occurred independently of any transposon activity.

In addition to relic presence/absence, the chromosome 3 region in Figure 4 was also polymorphic due to strain-to-strain differences in occupancy by other transposons (Figure 4). In many cases, these transposon insertions were “clean” with obvious target site duplications pointing to their acquisition via transposition. Conversely, none of the MoTeR relics had target site duplications, so there was no evidence that any of the internal relics arrived through transposition. Thus, the original MoTeR internalization events would have necessarily required the addition of chromosomal sequences at the 5′ ends of telomeric copies. This, in turn, would have generated a duplication or a translocation - either of which would have created new syntenic relationships. Therefore, to explain the absence of novel synteny in any of our strain comparisons, most internalization events must have occurred prior to the divergence of the various *P. oryzae* lineages.

### Interstitial Telomeres Are Generated Following Failures in Telomere Maintenance

The poor conservation of TJ and TAS reported above suggests that *P. oryzae* routinely experiences failures in telomere maintenance, which is predicted to cause replicative attrition and possibly exonuclease-mediated loss of terminal sequences. To explore the fates of deprotected chromosome ends in *P. oryzae*, we deleted the telomerase (TERT) gene from two lab strains, 60-3 and 2539, and characterized alterations in chromosome end structure, along with collateral genomic alternations. Both strains were generated through crosses between *P. oryzae* strains infecting different hosts (Leung et al., 1988; Starnes et al., 2012), such that some telomeres possess MoTeR insertions, while others lack them. In both strains, the deletion of TERT resulted in a period of telomere crisis characterized by a complete cessation of growth - a phenotype from which many strains never recovered. However, a small proportion of “survivor” strains eventually started re-growing, albeit slowly, and these were used to characterize alternative telomere maintenance mechanisms and the fates of the chromosome ends.

**FIGURE 5:**
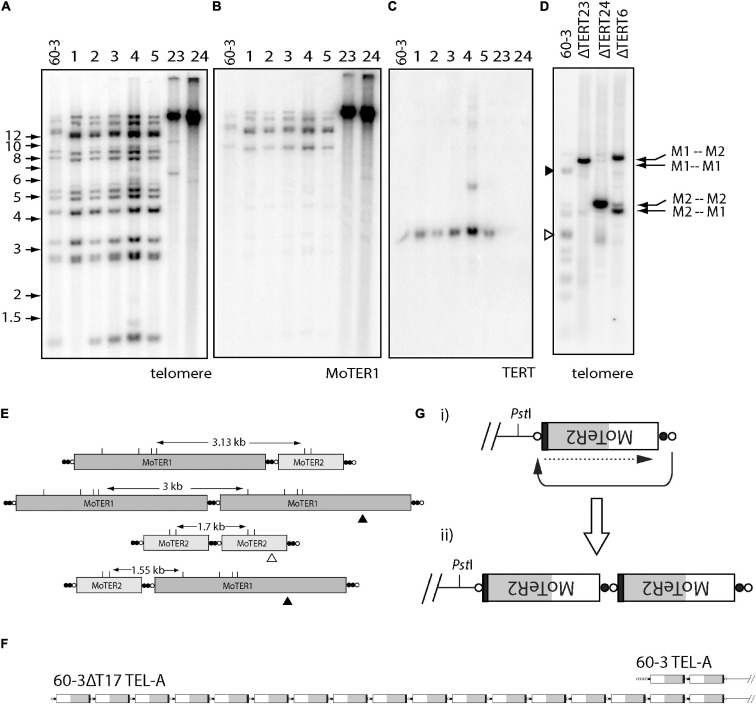
Alternative telomere maintenance (ATM) in telomerase knockout (TERT KO) mutants of strain 60-3. DNA was extracted from candidate telomerase knockout strains, digested with *Pst*I, size-fractionated by gel electrophoresis, and blotted to a membrane. Shown are phosphorimages obtained after sequential hybridization with PCR-generated, ^32^P-oligolabeled probes for panels **(A,D)** telomere repeats, **(B)** MoTeR1, and **(C)** TERT. Note the absence of parental telomeric fragments in the TERT KO isolates 60-3ΔT23 and 60-3ΔT 24; and their replacement by intensely hybridizing, high molecular weight fragments. **(C)** shows successful TERT deletion in strains 23 and 24; and **(D)** shows phosphorimage of a gel-fractionated *Mbo*I digest of DNA TERT KO strains 60-3ΔT6, 60-3ΔT23 and 60-3ΔT 24, after hybridization with the telomere probe. Again, note the absence of the parental telomeric fragments and their replacement by intensely hybridizing, yet discrete, *Mbo*I fragments, whose sizes correspond to specific MoTeR-MoTeR junctions **(E)**. **(E)** Diagram showing how different tandem MoTeR arrangements cause the interstitial telomeres to be contained on different-sized *Mbo*I fragments. Telomeric and telomere-like (present at MoTeR 5′ ends) sequences are shown as white and black circles, respectively; *Mbo*I sites are marked as “M.” **(F)** Example of MoTeR array amplification in TERT KO strain 60-3ΔT17, compared with the 60-3 parent strain. **(G)** Diagram showing how D-loop formation and telomere attrition-induced repair can result in the generation of tandemly amplified MoTeR arrays at the chromosome ends. The compromised telomere loops back and invades the interstitial telomere sequence (black arrow) and copies the distal sequence (dotted arrow). Repeated iteration of this process can generate extended arrays.

Telomerase reverse transcriptase KO lines derived from the strain 60-3 (Starnes et al., 2012), displayed dramatically altered telomere profiles in Southern blots, where the discrete telomeric *Pst*I fragments in the parent strain were replaced with high molecular weight bands whose hybridization intensities were significantly greater than the sum of the individual bands in the parent (Figure 5A). This pattern signals amplification of the telomere repeat sequences. The high molecular weight fragments also showed intense hybridization to a MoTeR1 probe (Figure 5B), pointing to concomitant amplification of MoTeR sequences. MoTeR elements lack *Pst*I sites such that digestion with this enzyme releases telomeric restriction fragments that contain entire MoTeR arrays. To explain the signal amplification and TRF size increases, we surmised that MoTeR sequences and the telomere repeats that separate them experienced tandem amplification at the chromosome ends. To confirm this, we blotted DNA samples that had been digested with *Mbo*I, which has sites in both MoTeR1 and MoTeR2, and then probed with the telomere repeat (Figure 5D). This produced strong hybridization signals at positions that correspond to *Mbo*I fragments expected for the four possible tandem MoTeR structures (M1-M1; M1-M2; M2-M1; and M2-M2) (Figure 5E). Tandem amplification of MoTeR arrays was confirmed using Nanopore sequencing data for two other 60-3 TERT KO strains (60-3ΔT15 & 17), which also revealed amplification of telomere plus MoTeR-derived, telomere-like sequence, separating each MoTeR copy - (CCCTAA)_2_(CCCGAA)_2_(CCCAAA)_8_CCCGAA. Several reads contained tandem MoTeR arrays that are much longer than any found in the parent strain, with a particularly good example, being a read showing the addition of at least 15 MoTeR2 copies to what was originally a two-element array in TEL-A on the left arm of minichromosome 1 (Figure 5F). Each MoTeR copy was separated from the next by short telomere tracts, which explains the increased intensity of telomeric hybridization signals in Figure 5A. Here, the number of repeats between MoTeR copies will initially depend on the length of the invading (compromised) telomere, the numbers of TTAGGG units in the target, and the register of the invasion event.

Based on the above result, we propose that amplification is initiated when replicative telomere attrition results in the loss of telomeric protein binding, which in turn allows the deprotected end to form a D-loop and invade interstitial telomere motifs between MoTeR copies. This would then initiate cycles of telomere attrition-induced replication (TAIR) to produce tandemly amplified arrays (Figure 5G). Other de-protected, MoTeR-containing chromosome ends could then capture the amplified array through additional TAIR events. Interestingly, in KO strains ΔT6 and ΔT23, the intense signals were at different positions, consistent with the preferential amplification of a different, MoTeR-MoTeR junction in the two strains (Figure 5D). These differences could be because the different TERT KO lines experienced propagation of amplified arrays that were initially established at different MoTeR-containing chromosome ends.

**FIGURE 6:**
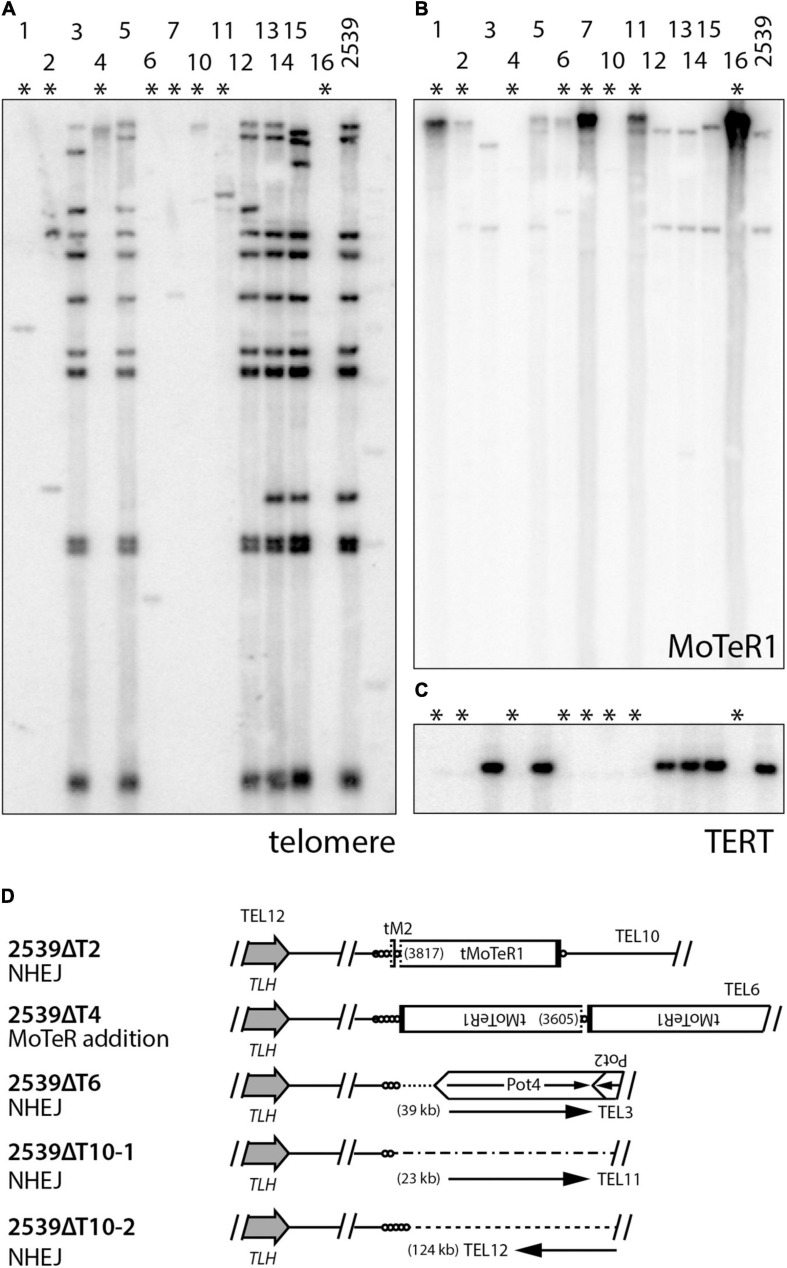
Alternative telomere maintenance (ATM) in telomerase knockout (TERT KO) mutants of strain 2539. DNA was extracted from candidate telomerase knockout strains, digested with *Pst*I, size-fractionated by gel electrophoresis, and blotted to a membrane. Shown are phosphorimages obtained after sequential hybridization with probes for panels **(A)** telomere repeats, **(B)** MoTeR1, and **(C)** TERT. **(D)** shows repair mechanisms for telomere 12 in select TERT KO strains. TLH = telomere-linked helicase gene; circles = telomere repeats (lengths not to scale); MoTeR = *M. oryzae* telomeric retrotransposon; tMoTeR = truncated element with dotted line showing truncation boundary and parentheses listing truncation positions; Pot2/4 = inverted repeat transposon; arrows show direction to nearest telomere and distances to telomere are listed in parentheses.

Parallel experiments in other *P. oryzae* strains indicate that the tandem amplification of subterminal sequences following TERT KO requires the presence of interstitial telomere motifs (manuscript in preparation). This begs the question: what are the fates of chromosome ends that lack such motifs? This was addressed using strain 2539 which has very few MoTeR copies in its genome. TERT deletions in 2539 produced KO strains showing almost complete losses of telomeric hybridization signals (Figure 6A). Furthermore, amplification of MoTeR sequences did not appear to be a dominant ATM mechanism (Figure 6B). To determine the fate of the 2539 telomeres, we used inverse PCR to amplify the sequences adjacent to TEL12 in a number of transformants. This telomere was chosen because it was known to lack MoTeR insertions and would presumably be repaired via a mechanism other than D-loop formation. TAS were successfully amplified from four different TERT KO lines, with 2539ΔT10 yielding two different amplicons. Sequencing revealed that a small telomere vestige was preserved in all cases (Supplementary Figure 2), and the compromised ends were repaired via two distinct repair pathways, with both using other subterminal sequences during the repair process (Figure 6C). 2539ΔT4 had acquired MoTeR sequences - most likely from another chromosome end through TAIR, or possibly via transposition. The other molecules were apparently repaired either by micro-homology mediated TAIR - a telomere-specific variant of micro-homology mediated break-induced replication (MMBIR; Hastings et al., 2009) in the case of 2539ΔT2, or by non-homologous end-joining (NHEJ). Having said this, it is not clear how NHEJ would have allowed telomere vestiges to be joined with sequences occurring at distances that are around 20 to more than 120 kb from a chromosome end (Figure 6D), as this would involve one chromosome end losing approximately 150 bp of sequence, and the other losing tens of kilobases.

### Spontaneous Telomere Failure Drives Amplification of Subtelomeric Sequences and Interstitial Telomere Formation in Foxtail Pathogens

**FIGURE 7:**
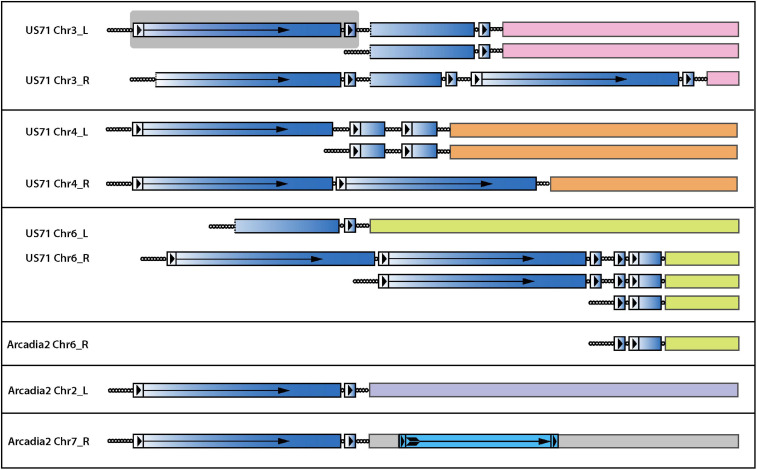
Interstitial telomere sequences define the boundaries of subtelomeric tandem repeats in the foxtail pathogens. The figure shows the organization of all interstitial telomeres identified in the Arcadia2 and US71 genomes. Full-length and partial copies of an associated tandem repeat are shaded with a blue gradient shading to illustrate the origin of partial repeat sequences and dotted lines indicate truncations ends. Long terminal repeat (LTR)-like structures are shown as boxes with arrowheads, and the reverse-transcriptase coding regions is marked with an arrow. A full-length repeat with LTRs is highlighted with a gray background. A bona-fide LTR retrotransposon insertion in Arcadia2 Chr7 is shown with lighter blue shading. Interstitial telomere sequences are shown as circles, with each circle representing approximately four CCCTAA repeat motifs. Truncated variants detected among MinION raw reads are illustrated beneath the respective main chromosome end structures.

In general, it is extremely rare to find telomeric sequence motifs with more than four CCCTAA/TTAGGG repeats at internal genome locations in *P. oryzae* - probably because long, interstitial telomere tracts experience frequent breakage and healing via telomere addition (Florea et al., 2016; Rahnama et al., 2020). *P. oryzae* strains from foxtails (*Setaria* spp.) are notable exceptions because they contain variable numbers of long, interstitial telomere sequences. Remarkably, strain US71 has eight, with lengths of 6, 9, 11, 16, 22, 27, 32, and 34 repeat units - the latter four being native telomere length. Arcadia has a single, 23-repeat internal array (Table 1). A clue to the origin and persistence of these highly labile motifs came from the observation that all instances reside near chromosome ends and are interspersed with, and form the boundary of, a 4 kb repeat sequence that occurs, along with variously truncated copies, in subtelomeric, tandem arrays (Figure 7). This arrangement is strikingly reminiscent of the tandem arrays that are generated in TERT KO strains (Figure 5F) which suggests that they too might have been generated during an episode of spontaneous telomere failure. Normally, interstitial telomeres should be resolved quite quickly by break-healing. Indeed, such events were readily detected near US71 TELs 5, 7, and 12 by aligning MinION reads to the chromosomal assembly and identifying groups of reads that had one end anchored in single copy sequence but then terminated in telomeric sequence at the interstitial telomere (Figure 7). To explain the persistence of multiple extended, internal repeats in these strains, we speculate that they “suffered” telomere failure quite recently; and possibly experience such events in an ongoing and periodic fashion.

**FIGURE 8:**
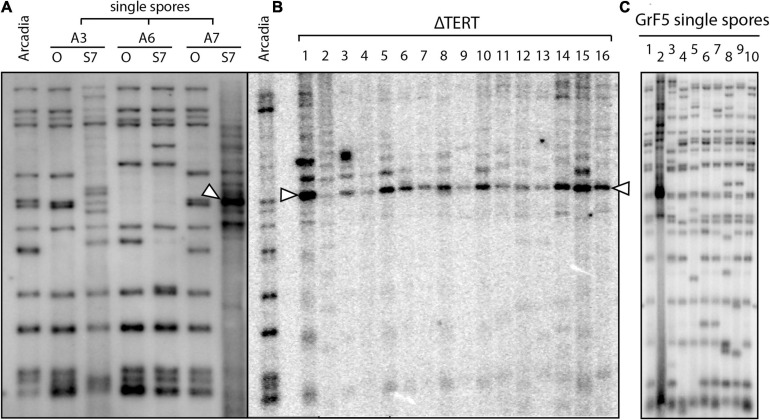
Spontaneous telomere failure in foxtail pathogens. **(A)** Single spores (SSs) were isolated from transformants of strain Arcadia2 that had been transformed with a MoTeR1 expression construct (Lane A). Three of the SS cultures (A3, A6, A7) were serially transferred on agar medium for 7 generations. Genomic DNA was extracted from the original (O) and 7th generation subcultures (S7), digested with *Pst*I and analyzed by Southern hybridization with a telomere probe. Note that the telomere profiles for the S7 cultures of strains A3 and A7 are very different to the profiles of the originals. S7 shows a typical Type I alternative telomere maintenance pattern (arrowhead), consistent with tandem amplification of the vestigial telomere plus the telomere-adjacent region (including the *Pst*I site). Note the faint background signals in the A3-S7 lane. This culture appears to be in the early stages of ATF, with several novel telomeres appearing. By comparison, S7 appears to have a more advanced ATF. **(B)** shows *Pst*I telomere fingerprints for 16 TERT KO strains in the wild-type Arcadia2 background. If one compares the profiles of the left lane (Arcadia2, A) with the A7/O lane, it appears that the fragment amplified in A7/S7 is the same as ones amplified in several TERT KO strains (arrowheads). **(C)** Variation in telomere fingerprint profiles among 10 single-spore cultures that were all isolates from a single parent culture that was also recently single-spored. Note the single, intense signal in culture 2 that indicates recent operation of an alternative telomere maintenance pathway.

The idea that interstitial telomere formation and subtelomere amplification occurs through ongoing episodes of telomere dysfunction is intriguing because it has long been proposed that spontaneous losses in telomere function may serve as a mechanism to spur genomic alterations with adaptive benefits (McEachern, 2008); and, while it has been shown that induced telomere failure can accelerate adaptation in an engineered evolution experiment (Mason and McEachern, 2018), until now, there have been no documented examples of spontaneous telomere failure in a native organism. As part of a study to investigate MoTeR transposition, we transformed a foxtail pathogen, Arcadia2, with a construct containing a copy of MoTeR1, and then screened single spore cultures for alterations in telomere profiles that would signal new insertions. Although MoTeR1 mobilization was not detected, we found that serial culture of one of the transformants (A7) resulted in the disappearance of parental telomere fragments, and the appearance of one band with a particularly intense hybridization signal (Figure 8A) - i.e., a pattern strikingly reminiscent of the telomere profiles seen in TERT KO strains (see Figures 5B,D). While we believe that this was a spontaneous telomere failure event and incidental to the transformation procedure, we cannot not rule out the possibility that it was precipitated by expression of the MoTeR1 transgene. However, a second, truly spontaneous telomere failure event was subsequently identified in a wild-type strain, GrF5-2, when a similar telomere profile showed up in a single-spore from a culture that had recently been purified by single-spore isolation (Figure 8C). Targeted cloning and sequencing of a representative fragment from the intensely hybridizing band revealed that it comprised the previously described tandem repeat bordered by interstitial telomeres (Supplementary Figure 3). Thus, we were able to associate tandem amplification of the subtelomeric repeat, with a spontaneous telomere failure event.

To obtain evidence that tandem amplification of the subtelomeric repeat is a direct response to telomere failure, we deleted the TERT gene from the wild-type Arcadia2 strain. Transformants with KOs were identified based on a sudden arrestation of growth and development of a characteristic hyphal “bubbling” phenotype. DNA was extracted from surviving transformants, and telomere fingerprinting was performed. All of the TERT KO strains showed losses of most parental telomere fragments and the appearance of intense signals with varying amplitudes at the same position as the band seen in the original Arcadia2 transformant (Figure 8B).

The 4 kb sequence codes for a predicted reverse transcriptase (RT) and is bordered by direct repeats. Thus, its structure and content resembles that of a long terminal repeat (LTR) retrotransposon. However, this resemblance is likely coincidental because the RT sequence is absent from most *P. oryzae* genomes and single-copy in most of the genomes where it is found. The repeat unit is also much shorter than typical fungal LTR-retrotransposons, and the RT gene lacks the integrase and RNase H domains required for transposition. In fact, a blastx search of the nr database revealed the only domain similarity to retrotransposon RTs was in the nucleotide binding region which, intriguingly, showed the closest similarity to telomerase RTs. Given that the 4 kb “element,” and its terminal repeats, are all bordered by telomere motifs (Figure 7 and Supplementary Figure 3), this suggests that it first originated following a telomere failure.

### Catastrophic Telomere Alterations Following a Spontaneous Gain of Virulence

**FIGURE 9:**
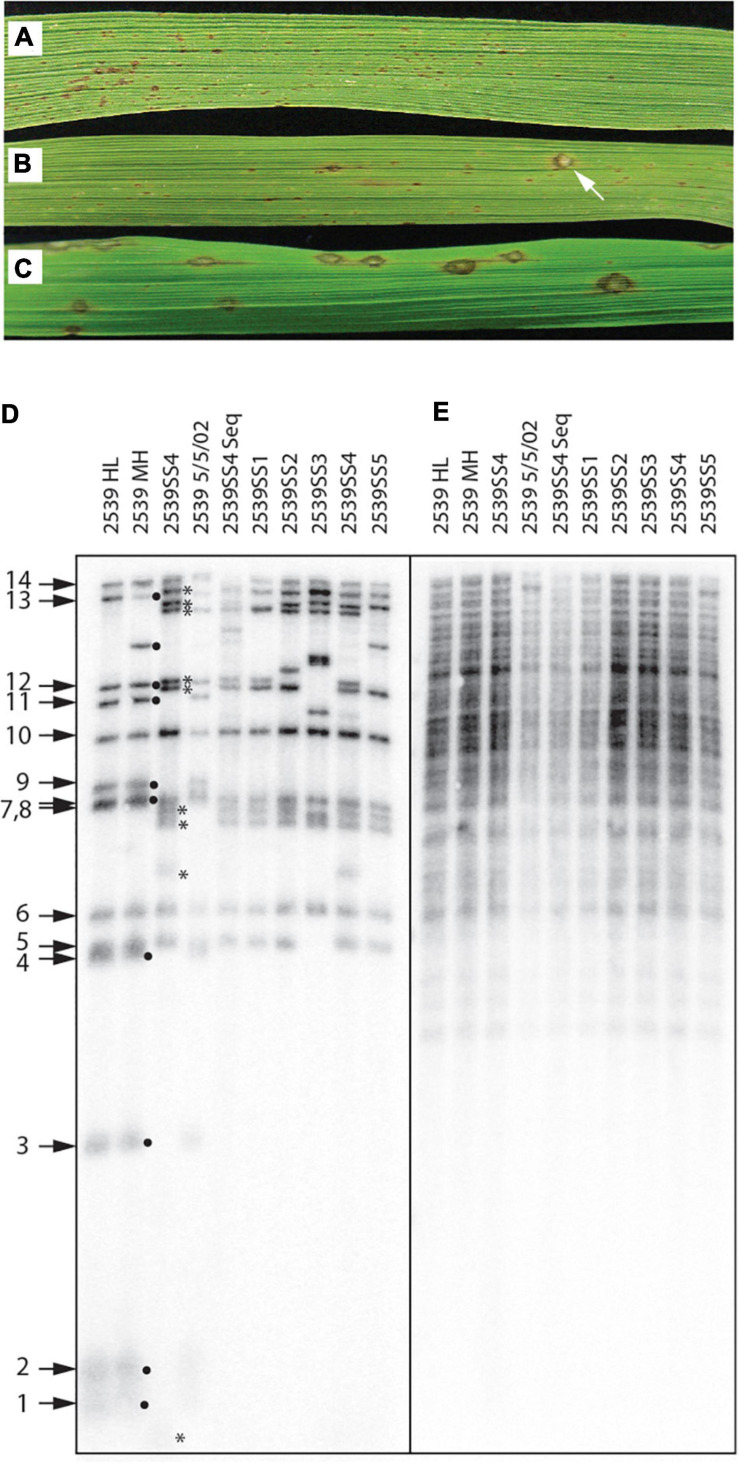
Telomere failure associated with an adaptive event. **(A)** Typical infection phenotype of avirulent strain, 2539, on rice cultivar 51583; **(B)** Gain of virulence for 2539 on 51583 as indicated by the presence of a rare single lesion; **(C)** Infection phenotype of a single-spore (SS) culture recovered from the lesion shown in panel **(B)** shows that the gain of virulence is stable; **(D)** Telomere fingerprints of single-spore cultures recovered from the lesions compared with several lab stocks of 2539 reveal alterations of telomeric fragments in the SS cultures recovered from the rare lesion; **(E)** DNA fingerprinting of the same strains shown in panel **(D)** using a hybridization probe that targets two dispersed repetitive elements (MGL and Pot2).

Direct evidence that telomere failure might provide an adaptive benefit came from an unrelated, long-term project where we were investigating the genetics of host specificity. As a control for that study, we would routinely inoculate strain 2539, on a rice cultivar (51583) that it is normally unable to infect (Figure 9A). In one particular experiment (out of dozens), we identified a single lesion on a single plant (Figure 9B). Spores were harvested from this lesion and used to establish a number of pure single-spore (SS) cultures. Inoculation of spores from a representative SS culture (2539ss4) on 51583 produced abundant lesions (Figure 9C), thereby confirming that the strain had acquired a stable gain of virulence.

Due to the uniqueness of this event, we initially suspected that the lesion might have been caused by cross contamination from a virulent progeny of 2539 that was inoculated in the same experiment. In an attempt to rule this out, we compared the telomere fingerprints of 2539ss4 with its parent, 2539MH. At first, this pointed to contamination because nine of the 14 telomeres in the parent culture were missing in 2539ss4 (Figure 9D, black dots), and nine novel fragments were observed in their place (asterisks). In contrast, various 2539 cultures stored at various times over the past 23 years had almost identical profiles to 2539MH, with the only exceptions being due to changes in rDNA and MoTeR-containing telomeres that are known to be unstable (Figure 9D).

Although F1 progeny are expected to share ∼50% of telomeric fragments with the 2539 parent, none of the novel fragments matched telomeres in the other parent used in the cross (data not shown). Therefore, we were unconvinced that the 2539ss4 culture was a contaminant and proceeded to strip the blot and re-probe it with a highly repetitive DNA fingerprinting probe. All cultures had virtually identical fingerprints (Figure 9E), which would not be expected if the 2539ss cultures had inherited 50 % of their genomes from a distantly related strain. Finally, a genome assembly was generated for 2539ss4 and comparison with 2539 indicated that they were clones of one another because the SNP frequency (3.6E-5) was well within typical SNP calling error rates (Farman and Ramachandran, unpublished data). This absolutely ruled out the possibility of contamination, and lead to the conclusion that the dramatic alteration of telomere profiles in the 2539ss cultures must have resulted from a temporary failure in telomere maintenance. Here, it should be noted that intense telomere signals are not expected following spontaneous telomere failure in 2539 because these were not observed in the TERT KO experiments (Figure 6A).

## Discussion

It is generally accepted that the subterminal chromosome regions are the most dynamic of genome compartments (Mefford and Trask, 2002; Linardopoulou et al., 2005; Chen et al., 2018). It is rather surprising, therefore, that there are very few reports where chromosome ends have been “caught in the act” of changing (Horowitz et al., 1984; Möller et al., 2018; Coulon and Vaurs, 2020). *P. oryzae* is perhaps the champion of telomere dynamicism because, in the most extreme cases, telomere alterations can be found in every single-spore culture established from a genetically purified colony (Starnes et al., 2012); and interrogation of MinION sequence data identified telomere rearrangements in 60% of the chromosomal molecules surveyed (Rahnama et al., 2020). Early experiments suggested that the most extreme telomere instability was restricted to telomeres containing MoTeR insertions (Starnes et al., 2012) but subsequent experiments monitoring telomere alterations in single-spore cultures revealed several exceptional strains – most notably from foxtails – that also showed rampant telomere instability, despite the fact that they lacked telomeric MoTeRs (Farman et al., 2014). Here, we used comparative genomics to explore telomere instability at a population level, and to gain insights into the genetic basis for extreme sequence variation at the chromosome ends of different *Pyricularia* strains.

One of the primary presumed functions of telomeres is to protect the chromosome ends from sequence loss due to replicative attrition, or exonucleolytic attack (Zakian, 1989). If they functioned perfectly, the telomere structure at any given chromosome end should be identical among isolates - the repeats should be attached to the same internal sequence, with the only variation being due to nucleotide substitutions/indels/transposon insertions within the constituent sequences. However, our examination of a comprehensive collection of *Pyricularia* strains, revealed that telomere structure was rarely preserved, and it was extremely rare to find a specific TJ in more than a handful of isolates. So, even though many *Pyricularia* strains exhibited few, if any, telomere alterations in clonal propagation experiments (Farman et al., 2014), it is clear that genetic instability at the chromosome ends is ubiquitous within the genus, and occurs frequently in nature.

Not surprisingly, the poor TJ preservation translated to poor conservation of TASs, as might be expected if entire chromosome ends had been lost. However, there were also many cases where a TAS identified in one strain was present in other strains but resided in the genome interior. By mapping these TASs to a common reference genome, we gained insights into the evolutionary histories of the chromosome ends. Mapping of some TASs revealed a pattern of nested deletions (e.g., Figure 2C) that pointed to the various strains having experienced occasional bouts of mild telomere failure that resulted in progressive chromosome truncations with eventual *de novo* telomere formation. Indeed, this is the most likely explanation for the truncations that gave rise to *P. oryzae* mutants lacking *Avr-Pita* (Orbach et al., 2000). Of course, an alternative explanation for the poor conservation and chromosomal organizations of TJs and associated TAS is that they are co-casualties of frequent breaks at internal chromosome positions that result in deletion of the chromosome ends. However, this seems unlikely because chromosome breakage normally causes cell cycle arrest (Sandell and Zakian, 1993), which means that repeated, spontaneous breakages would need to occur at different positions on the same chromosome to account for the nested truncation patterns. Additionally, internal breaks cannot explain the origins of interstitial telomere sequences because the telomeric ends of the released fragments would be ineligible to interact with other genomic sequences until they too were deprotected. On the other hand, we have shown that both telomeres and MoTeR sequences experience resection/truncation and internalization during the repair of experimentally induced deprotected telomeres (Figure 6D).

In fact, by analyzing the fates of just a few telomeres in a single survivor from one TERT KO experiment, we were able to document a range of repair outcomes (Figure 6D) which, along with collateral events (such as breaks in interstitial telomeres), could fully account for the content and organization of the *P. oryzae* subterminal chromosome regions, and their variation among strains/species. The first key finding was that former telomeres were internalized because their repair resulted in the capture of sequences found in other subterminal regions (Figure 6). Second, repair was usually duplicative, such that other chromosomal sequences were copied, and not translocated, onto the de-protected chromosome ends. Thus, telomere failure and ensuing repair not only provides a plausible explanation for why and how telomeres/MoTeR relics/TAS become internalized, but also provides a mechanistic basic for their tendency to end up in subterminal regions, and associated with duplications. That these processes also operate under spontaneous telomere failure is supported by our prior documentation of spontaneous telomere alterations that resulted in TAS internalizations via duplicative capture of sequences from subterminal locations (Rehmeyer et al., 2006; Starnes et al., 2012).

This study is the first to explore the chromosomal landscapes of features that used to occupy telomeric locations but have since immigrated to the genome interior. When viewed in the light of demonstrated responses to telomere failure, these features provide abundant evidence of both historical and on-going interactions between the telomeres and sequences in the genome interior and, in doing so, suggest a mechanistic basis for chromosomal domain structure. Regardless of the dynamic surveyed - TAS immigrations/emigrations; MoTeR/telomere relic internalizations; relic-associated duplications; or the capture of internal sequences during telomere repair - the flow and sharing of sequence information was largely restricted to the subterminal portions of the chromosomes. This, along with our finding that telomere repair captures subterminal sequences in an apparently indiscriminant manner, implies that different *P. oryzae* telomeres/subtelomeres/subterminal regions tend to be clustered within the nucleus, as has been shown for other organisms (Gotta et al., 1996; Figueiredo et al., 2002; Batté et al., 2017). Telomeric clustering is likely to promote the adaptive benefits of spontaneous telomere failure by restricting telomeric “contacts” to chromosomal neighborhoods where architectural preservation is not so critical, and in doing so, may set up a situation in which almost anything goes in terms of telomere repair mechanisms, as long as what happens in the subterminal regions, stays in the subterminal regions.

Already, by mapping telomeric features onto just one reference genome, we have started to define subterminal chromosomal domains that have been impacted by historical telomere interactions - most likely as a consequence of spontaneous telomere loss. It is probably not coincidental that these domains are also hotspots for breakdowns in strain-to-strain chromosome synteny (Farman, personal observations). Comparative genome studies in yeast have also identified a distinct domain structure consisting of highly syntenic cores, which then transition into highly variable subtelomeric domains that exhibit complex rearrangements and segmental duplications (Yue et al., 2017). Based on our findings, it is tempting to speculate that much of the chromosomal (re-)organization (and duplications) in these domains is driven directly, or indirectly, by spontaneous telomere failure, with the domain boundaries being determined by how far de-protected ends can “reach” beyond the telomeric clusters in the nucleus.

Our detection of spontaneous telomere failure in at least two, and possibly three, different strains of a native organism is a landmark discovery. While, it has long been proposed that bouts of mild telomere dysfunction could be an adaptive strategy that contributes to the unusual structure and dynamicism of subtelomere regions (McEachern, 2008; Mason and McEachern, 2018), until now there had been no documented examples of “adaptive telomere failure” (ATF) in a native organism. Formally stated, the ATF hypothesis posits that:

“certain environmental stresses can induce a low level of telomere failure, potentially leading to elevated subtelomeric recombination that could result in adaptive mutational changes within subtelomeric genes” (Mason and McEachern, 2018).

It was further reasoned that, if it occurs, ATF should be mild so as to avoid cellular senescence, while sufficiently effective to cause occasional telomere de-capping. Counter to this prediction, we find that spontaneous telomere failure in *P. oryzae* can be very severe - to the extent that all parental telomeres were compromised in the foxtail pathogens, and more than 50% of telomeres were altered in strain 2539. This is perhaps fortuitous because if, by necessity, adaptive telomere failure should be so infrequent and mild as to prevent detrimental cellular phenotypes, one might expect the genomic effects to be correspondingly rare and mild and, therefore, virtually impossible to detect.

The foxtail pathogen lineage is clearly exceptional because member strains are unique in possessing abundances of long, interstitial telomere sequences and all indications are that these persist because telomere failure recurs quite frequently. On the other hand, only two telomeres in 2539 normally show re-arrangement with extended culturing (the rDNA telomere and one containing MoTeRs), so the detection of alterations at seven additional chromosome ends demonstrates that even strains with “stable” telomeres certainly can experience occasional, yet catastrophic, telomere re-arrangements. This lends further weight to our argument that the TJ/TAS variation in *Pyricularia*, internalization of telomeric entities, and the broader organization of the subterminal chromosome regions, is driven in large part by spontaneous telomere failures. These could either be rare and severe (affecting multiple telomeres at once), or fairly frequent and affecting just a few telomeres at a time (i.e., in a similar fashion to rDNA instability, Farman et al., 2014; Rahnama et al., 2020).

Curiously, there was no indication that the three strains experienced a period of “telomere crisis” that accompanies experimentally induced telomere loss (Zlotorynski, 2019) (and this study). This, and the fact that most *P. oryzae* strains exhibit stable telomere profiles in between projected episodes of spontaneous failure, suggests that the physiological state that promotes telomere loss is usually temporary. Mild failures could be stochastic and occur through occasional insufficiency of one or more TERT holoenzyme components, while severe bouts might be driven by epigenetic silencing of these components, or of telomere capping proteins. Indeed, failed telomere capping would appear to be the most plausible explanation for the long (native telomere-length) interstitial telomeres in the foxtail pathogens. Lastly, it is also possible that initial telomere shortening results from the illegitimate activation of a process known as Telomere Rapid Deletion, that is believed to be involved in telomere length homeostasis (Lustig, 2003; Watson and Shippen, 2007).

Spontaneous telomere failure has the potential to confer direct and immediate adaptive benefits. In *P. oryzae*, the initial resection of chromosome ends has the potential to evict genes that restrict the pathogen’s host range, as was previously demonstrated for *AVR-Pita* (Orbach et al., 2000). It is possible that such an occurrence may underlie 2539’s gain of virulence on cultivar 51583. Next, the processes used to repair the resected ends can drive immediate adaptation through a variety of mechanisms. First, as we have shown for MoTeRs in 60-3, and the 4 kb (RT) repeat in the foxtail pathogens, the presence of short telomere tracts in the subterminal regions allows for massive tandem amplification of genes in the intervening region - presumably via repeated rounds of TAIR, rolling circle replication (Lustig, 2003), or replication of extrachromosomal circle templates (Natarajan and McEachern, 2002). Whether or not the amplification of these specific sequences serve adaptive purposes will require elucidation through functional assays. If anything, we might expect increased MoTeR RT expression to have a negative impact by interfering further with telomere function by virtue of its predicted telomere cleavage activity (Starnes et al., 2012). On the other hand, the 4 kb repeat’s resemblance to LTR retrotransposons is likely coincidental, and the RT gene may instead belong to a class of “RVT” proteins, which may have global effects on cellular RNA processes, through RNA-dependent RNA polymerase activity (Gladyshev and Arkhipova, 2011). The potential for subterminal tandem amplification to drive adaptation is nicely illustrated by the *Cryptococcus neoformans* arsenite transporter gene, *ARR3*, which occurs in a large tandem array in the subterminal region at the right end of chromosome 3, and is associated with enhanced arsenic tolerance (Chow et al., 2012). Based on our findings, we speculate that amplification of *ARR3* arrays is an outcome of adaptive telomere failure.

In the absence of subterminal telomeric tracts, ATM in *P. oryzae* involves the recruitment of sequences from the genome interior. The adaptive benefits of this dynamic are two-fold: First, because these events are usually duplicative, depending on the length of copied sequence, this can lead to the duplication of many tens of genes, with the possibility of enhancing a wide variety of adaptive traits (Kondrashov, 2012). Second, by copying or moving genes from relatively static internal chromosome regions to the highly dynamic chromosomes ends, those genes can now enjoy the enhanced evolutionary environment and, if this serves some adaptive benefit, they may persist there. There are manifold examples of niche-adaptation genes accumulating in subtelomeric regions (Charron et al., 1989; Denayrolles et al., 1997; Barry et al., 2003) but until now has not been a satisfactory mechanistic explanation for how they find their way to the chromosome locations best suited for adaptive exploration. The capture of internal sequences during the repair of spontaneously compromised chromosome ends would provide such a route.

Lastly, spontaneous telomere failure can be an adaptive gift that keeps on giving. In addition to generating immediate chromosome alterations, repair of compromised telomeres can lead to the creation of unstable structures that promote ongoing genomic change. Prime examples are the long interstitial telomeres in the foxtail pathogens which experience breakage at high frequencies, and generate substrates that undergo telomerase-mediated healing (e.g., US71 Chr6, Figure 7), and might also participate in further rearrangements. The potential for interstitial telomeres to have long-term impacts on genome structure is amply illustrated by the MoTeR relics, which have variable length telomere tracts that their 3’ ends (Rahnama et al., 2020). A comparative analysis of relic polymorphism uncovered a multitude of intrachromosomal deletions centered around the relic loci. This is most economically explained by repeated, independent evictions of the relics and varying amounts of flanking DNA, having been initiated by breaks in the associated interstitial telomeres, followed by resections, and eventual repair by NHEJ. Finally, although we have not yet detected, or explored, such occurrences in *Pyricularia*, telomere repair has the potential to unleash a cascade of genomic change through breakage-fusion-bridge cycles (Lo et al., 2002), and these are almost certainly additional factors that contribute to the dramatic shuffling of sequences that can often be found in the subterminal regions (Farman et al., 2014; Yue et al., 2017).

## Data Availability Statement

Assembled genome sequences and raw sequence reads utilized in this project are available at NCBI (https://www.ncbi.nlm.nih.gov) under the following BioProjects: PRJNA320483 and PRJNA579424.

## Author Contributions

MR generated and analyzed the MinION sequence data, wrote software, generated most of the plots and data tables, and was involved in manuscript editing and submission. BW generated, identified, and characterized 2539 TERT KO strains. JD analyzed the MoTeR relic distributions and duplications and wrote sections of the manuscript. ON generated and analyzed single-spore isolates for foxtail pathogens. DY characterized TEL12 repair events in 2539. AY performed Southern blots of Arcadia TERT KO strains. HB, MB, and HJ analyzed the telomere junctions and telomere-adjacent sequences. XZ and JL performed the SeqOthello runs. AL discovered the spontaneous telomere rearrangements in the 2359ss cultures. AS wrote software that was integral to comparative genome analysis. MH identified the original 2539ss mutant. JH performed the first experiments that revealed spontaneous telomere failure. LC characterized strains showing spontaneous telomere failure. PC supervised the research of HB, MB, and HJ and contributed sections to the manuscript. MF conceived most experiments, directed the research, performed some experiments and bioinformatic analyses, and wrote most of the manuscript. All authors contributed to the article and approved the submitted version.

## Conflict of Interest

The authors declare that the research was conducted in the absence of any commercial or financial relationships that could be construed as a potential conflict of interest.

## Publisher’s Note

All claims expressed in this article are solely those of the authors and do not necessarily represent those of their affiliated organizations, or those of the publisher, the editors and the reviewers. Any product that may be evaluated in this article, or claim that may be made by its manufacturer, is not guaranteed or endorsed by the publisher.

## Footnotes

1
https://github.com/tseemann/VelvetOptimiser

2
https://github.com/drdna/iSNPcaller

3
https://github.com/drdna/TelEvolution

## References

[B1] Anderson M. Z. Wigen L. J. Burrack L. S. Berman J. (2015). Real-time evolution of a subtelomeric gene family in *Candida albicans*. *Genetics* 200 907–919. 10.1534/genetics.115.177451 25956943 PMC4512551

[B2] Barry J. D. Ginger M. L. Burton P. McCulloch R. (2003). Why are parasite contingency genes often associated with telomeres? *Int. J. Parasitol.* 33 29–45. 10.1016/s0020-7519(02)00247-3 12547344

[B3] Batté A. Brocas C. Bordelet H. Hocher A. Ruault M. Adjiri A. (2017). Recombination at subtelomeres is regulated by physical distance, double-strand break resection and chromatin status. *EMBO J.* 36 2609–2625. 10.15252/embj.201796631 28754657 PMC5579382

[B4] Berriman M. Ghedin E. Hertz-Fowler C. Blandin G. Renauld H. Bartholomeu D. C. (2005). The genome of the African trypanosome *Trypanosoma brucei*. *Science* 309 416–422. 10.1126/science.1112642 16020726

[B5] Bonman J. M. (1992). Durable resistance to rice blast disease – environmental influences. *Euphytica* 63 115–123. 10.1007/978-94-017-0954-5_10

[B6] Cervenak F. Sepsiova R. Nosek J. Tomaska L. (2021). Step-by-Step evolution of telomeres: lessons from yeasts. *Genome Biol. Evol.* 13:evaa268. 10.1093/gbe/evaa268 PMC7857110 33537752

[B7] Charron M. J. Read E. Haut S. R. Michels C. A. (1989). Molecular evolution of the telomere-associated *MAL* loci of *Saccharomyces*. *Genetics* 122 307–316. 10.1093/genetics/122.2.307 2548922 PMC1203703

[B8] Chen N. W. G. Thareau V. Ribeiro T. Magdelenat G. Ashfield T. Innes R. W. (2018). Common bean subtelomeres are hot spots of recombination and favor resistance gene evolution. *Front. Plant Sci.* 9:1185. 10.3389/fpls.2018.01185 30154814 PMC6102362

[B9] Chow E. W. L. Morrow C. A. Djordjevic J. T. Wood I. A. Fraser J. A. (2012). Microevolution of *Cryptococcus neoformans* driven by massive tandem gene amplification. *Mol. Biol. Evol.* 29 1987–2000. 10.1093/molbev/mss066 22334577

[B10] Coulon S. Vaurs M. (2020). Telomeric transcription and telomere rearrangements in quiescent cells. *J. Mol. Biol.* 432 4220–4231. 10.1016/j.jmb.2020.01.034 32061930

[B11] de Lange T. (2005). Shelterin: the protein complex that shapes and safeguards human telomeres. *Genes Dev.* 19 2100–2110. 10.1101/gad.1346005 16166375

[B12] Denayrolles M. de Villechenon E. P. Lonvaud-Funel A. Aigle M. (1997). Incidence of *SUC-RTM* telomeric repeated genes in brewing and wild wine strains of *Saccharomyces*. *Curr. Genet.* 31 457–461. 10.1007/s002940050230 9211787

[B13] Donelson J. E. (2003). Antigenic variation and the African trypanosome genome. *Acta Trop.* 85 391–404. 10.1016/s0001-706x(02)00237-1 12659976

[B14] Edgar R. C. (2004). MUSCLE: a multiple sequence alignment method with reduced time and space complexity. *BMC Bioinformatics* 5:113. 10.1186/1471-2105-5-113 15318951 PMC517706

[B15] Fabre E. Muller H. Therizols P. Lafontaine I. Dujon B. Fairhead C. (2004). comparative genomics in hemiascomycete yeasts: evolution of sex, silencing, and subtelomeres. *Mol. Biol. Evol.* 22 856–873. 10.1093/molbev/msi070 15616141

[B16] Farman M. Novikova O. S. Starnes J. H. Thorbury D. W. (2014). “Subtelomere organization, evolution, and dynamics in the rice blast fungus *Magnaporthe oryzae*,” in *Subtelomeres*, eds Louis E. J. Becker M. M. (Berlin: Springer-Verlag), 71–99. 10.1007/978-3-642-41566-1_4

[B17] Farman M. L. (2007). Telomeres in the rice blast fungus: the world of the end as we know it. *FEMS Microbiol. Lett.* 273 125–132. 10.1111/j.1574-6968.2007.00812.x 17610516

[B18] Farman M. L. Kim Y.-S. (2005). Telomere hypervariability in *Magnaporthe oryzae*. *Mol. Plant Pathol.* 6 287–298. 10.1111/j.1364-3703.2005.00285.x 20565657

[B19] Figueiredo L. M. Freitas-Junior L. H. Bottius E. Olivo-Marin J. C. Scherf A. (2002). A central role for *Plasmodium falciparum* subtelomeric regions in spatial positioning and telomere length regulation. *EMBO J.* 21 815–824. 10.1093/emboj/21.4.815 11847128 PMC125872

[B20] Figueiredo L. M. Janzen C. J. Cross G. A. M. (2008). A histone methyltransferase modulates antigenic variation in African trypanosomes. *PLoS Biol.* 6:e161. 10.1371/journal.pbio.0060161 18597556 PMC2443197

[B21] Flanagan P. R. Fletcher J. Boyle H. Sulea R. Moran G. P. Sullivan D. J. (2018). Expansion of the *TLO* gene family enhances the virulence of *Candida* species. *PLoS One* 13:e0200852. 10.1371/journal.pone.0200852 30028853 PMC6054389

[B22] Florea S. Phillips T. D. Panaccione D. G. Farman M. L. Schardl C. L. (2016). Chromosome-end knockoff strategy to reshape alkaloid profiles of a fungal endophyte. *G3* 6 2601–2610. 10.1534/g3.116.029686 27334939 PMC4978913

[B23] Freitas-Junior L. Bottius E. Pirrit L. A. Deitsch K. W. Scheidig C. Guinet F. (2000). Frequent ectopic recombination of virulence factor genes in telomeric chromosome clusters of *P. falciparum*. *Nature* 407 1018–1022. 10.1038/35039531 11069183

[B24] Freitas-Junior L. H. Hernandez-Rivas R. Ralph S. A. Montiel-Condado D. Ruvalcab-Salazar O. K. Rojas-Meza A. P. (2005). Telomeric heterochromatin propagation and histone acetylation control mutually exclusive expression of antigenic variation genes in malaria parasites. *Cell* 121 25–36. 10.1016/j.cell.2005.01.037 15820676

[B25] Gardner M. J. Hall N. Fung E. White O. Berrlman M. Hyman R. W. (2002). Genome sequence of the human malaria parasite *Plasmodium falciparum*. *Nature* 419 498–511. 12368864 10.1038/nature01097 PMC3836256

[B26] Gladieux P. Condon B. Ravel S. Soanes D. Maciel J. L. N. Nhani A. Jr. (2018). Gene flow between divergent cereal- and grass-specific lineages of the rice blast fungus *Magnaporthe oryzae*. *mBio* 9:e01219-17. 10.1128/mBio.01219-17 PMC5829825 29487238

[B27] Gladyshev E. A. Arkhipova I. R. (2011). A widespread class of reverse transcriptase-related cellular genes. *Proc. Natl. Acad. Sci. U.S.A.* 108:20311. 10.1073/pnas.1100266108 21876125 PMC3251080

[B28] Gotta M. Laroche T. Formenton A. Maillet L. Scherthan H. Gasser S. M. (1996). The clustering of telomeres and colocalization with Rap1, Sir3, and Sir4 proteins in wild-type *Saccharomyces cerevisiae*. *J. Cell. Biol.* 134 1349–1363. 10.1083/jcb.134.6.1349 8830766 PMC2121006

[B29] Greider C. W. Blackburn E. H. (1989). A telomeric sequence in the RNA of *Tetrahymena* telomerase required for telomere repeat synthesis. *Nature* 337 331–337. 10.1038/337331a0 2463488

[B30] Hastings P. J. Ira G. Lupski J. R. (2009). A microhomology-mediated break-induced replication model for the origin of human copy number variation. *PLoS Genet.* 5:e1000327. 10.1371/journal.pgen.1000327 19180184 PMC2621351

[B31] Hazelhurst S. Hide W. Lipták Z. Nogueira R. Starfield R. (2008). An overview of the wcd EST clustering tool. *Bioinformatics* 24 1542–1546. 10.1093/bioinformatics/btn203 18480101 PMC2718666

[B32] Horowitz H. Thorburn P. Haber J. E. (1984). Rearrangements of highly polymorphic regions near telomeres of *Saccharomyces cerevisiae*. *Mol. Cell. Biol.* 4 2509–2517. 10.1128/mcb.4.11.2509-2517.1984 6392854 PMC369082

[B33] Keely S. P. Renauld H. Wakefield A. E. Cushion M. T. Smulian A. G. Fosker N. (2005). Gene arrays at *Pneumocystis carinii* telomeres. *Genetics* 170 1589–1600. 10.1534/genetics.105.040733 15965256 PMC1449779

[B34] Kondrashov F. A. (2012). Gene duplication as a mechanism of genomic adaptation to a changing environment. *Proc. R. Soc. Lond. B Biol. Sci.* 279 5048–5057. 10.1098/rspb.2012.1108 22977152 PMC3497230

[B35] Koren S. Walenz B. P. Berlin K. Miller J. R. Bergman N. H. Phillippy A. M. (2017). Canu: scalable and accurate long-read assembly via adaptive k-mer weighting and repeat separation. *Genome Res.* 27 722–736. 10.1101/gr.215087.116 28298431 PMC5411767

[B36] Krzywinski M. Schein J. Birol I. Connors J. Gascoyne R. Horsman D. (2009). Circos: an information aesthetic for comparative genomics. *Genome Res.* 19 1639–1645. 10.1101/gr.092759.109 19541911 PMC2752132

[B37] Kumar S. Stecher G. Li M. Knyaz C. Tamura K. (2018). MEGA X: molecular evolutionary genetics analysis across computing platforms. *Mol. Biol. Evol.* 35 1547–1549. 10.1093/molbev/msy096 29722887 PMC5967553

[B38] Kurtz S. Phillippy A. Delcher A. L. Smoot M. Shumway M. Antonescu C. (2004). Versatile and open software for comparing large genomes. *Genome Biol.* 5:R12. 10.1186/gb-2004-5-2-r12 PMC395750 14759262

[B39] Leech J. H. Barnwell J. W. Miller L. H. Howard R. J. (1984). Identification of a strain-specific malarial antigen exposed on the surface of *Plasmodium falciparum*-infected erythrocytes. *J. Exp. Med.* 159 1567–1575. 10.1084/jem.159.6.1567 6374009 PMC2187322

[B40] Leung H. Borromeo E. S. Bernardo M. A. Notteghem J. L. (1988). Genetic analysis of virulence in the rice blast fungus *Magnaporthe grisea*. *Phytopathology* 78 1227–1333.

[B41] Li H. (2018). Minimap2: pairwise alignment for nucleotide sequences. *Bioinformatics* 34 3094–3100. 10.1093/bioinformatics/bty191 29750242 PMC6137996

[B42] Li W. Rehmeyer C. J. Staben C. Farman M. L. (2005). TruMatch–a BLAST post-processor that identifies bona fide sequence matches to genome assemblies. *Bioinformatics* 21 2097–2098. 10.1093/bioinformatics/bti257 15671115

[B43] Linardopoulou E. V. Williams E. M. Fan Y. Friedman C. Young J. M. Trask B. J. (2005). Human subtelomeres are hot spots of interchromosomal recombination and segmental duplication. *Nature* 437 94–100. 10.1038/nature04029 16136133 PMC1368961

[B44] Lo A. W. I. Sabatier L. Fouladi B. Pottier G. Ricoul M. Murnane J. P. (2002). DNA amplification by breakage/fusion/bridge cycles initiated by spontaneous telomere loss in a human cancer cell line. *Neoplasia (New York, N.Y.)* 4 531–538. 10.1038/sj.neo.7900267 12407447 PMC1503667

[B45] Louis E. J. (2014). “Introduction,” in *Subtelomeres*, eds Louis E. J. Becker M. M. (Berlin: Springer Verlag), 1–12. 10.1093/actrade/9780199589944.003.0001

[B46] Lustig A. J. (2003). Clues to catastrophic telomere loss in mammals from yeast telomere rapid deletion. *Nat. Rev. Genet.* 4 916–923. 10.1038/nrg1207 14634639

[B47] Mason J. M. O. McEachern M. J. (2018). Chromosome ends as adaptive beginnings: the potential role of dysfunctional telomeres in subtelomeric evolvability. *Curr. Genet.* 64 997–1000. 10.1007/s00294-018-0822-z 29589105

[B48] McEachern M. J. (2008). “Telomeres: guardians of genomic integrity or double agents of evolution?,” in *Origins and Evolution of Telomeres*, eds Noseck J. Tomaska L. (Georgetown, TX: Landes Bioscience).

[B49] Mefford H. C. Trask B. J. (2002). The complex structure and dynamic evolution of human subtelomeres. *Nat. Rev. Genet.* 3 91–102. 10.1038/nrg727 11836503

[B50] Möller M. Habig M. Freitag M. Stukenbrock E. H. (2018). Extraordinary genome instability and widespread chromosome rearrangements during vegetative growth. *Genetics* 210 517–529. 10.1534/genetics.118.301050 30072376 PMC6216587

[B51] Natarajan S. McEachern M. J. (2002). Recombinational telomere elongation promoted by DNA circles. *Mol. Cell. Biol.* 22 4512–4521. 10.1128/mcb.22.13.4512-4521.2002 12052861 PMC133910

[B52] Naumova E. S. Naumov G. I. Masneuf-Pomarède I. Aigle M. Dubourdieu D. (2005). Molecular genetic study of introgression between *Saccharomyces bayanus* and *S. cerevisiae*. *Yeast* 22 1099–1115. 10.1002/yea.1298 16240458

[B53] Naumova E. S. Naumov G. I. Michailova Y. V. Martynenko N. N. Masneuf-Pomarède I. (2011). Genetic diversity study of the yeast *Saccharomyces bayanus* var. *uvarum* reveals introgressed subtelomeric *Saccharomyces cerevisiae* genes. *Res. Microbiol.* 162 204–213. 10.1016/j.resmic.2010.09.023 21112388

[B54] Nierman W. C. Pain A. Anderson M. J. Wortman J. R. Kim H. S. Arroyo J. (2005). Genomic sequence of the pathogenic and allergenic filamentous fungus *Aspergillus fumigatus*. *Nature* 438 1151–1156. 16372009 10.1038/nature04332

[B55] Orbach M. J. Farrall L. Sweigard J. A. Chumley F. G. Valent B. (2000). A telomeric avirulence gene determines efficacy for the rice blast resistance gene Pi-ta. *Plant Cell* 12 2019–2032. 10.2307/3871102 11090206 PMC152363

[B56] Pays E. Delauw M. F. van Assel S. Laurent M. Vervoort T. Van Miervenne N. (1983). Modifications of a *Trypanosome b. brucei* antigen gene repertoire by different recombinational mechanisms. *Cell* 35 721–731. 10.1016/0092-8674(83)90105-8 6197182

[B57] Peska V. Garcia S. (2020). Origin, diversity, and evolution of telomere sequences in plants. *Front. Plant Sci.* 11:117. 10.3389/fpls.2020.00117 32153618 PMC7046594

[B58] Peyyala R. Farman M. L. (2006). *Magnaporthe oryzae* isolates causing gray leaf spot of perennial ryegrass possess a functional copy of the *AVR1-CO39* avirulence gene. *Mol. Plant Pathol.* 7 157–165. 10.1111/j.1364-3703.2006.00325.x 20507436

[B59] Pryde F. E. Gorham H. C. Louis E. J. (1997). Chromosome ends: all the same under their caps? *Curr. Opin. Genet. Dev.* 7 822–828. 10.1016/s0959-437x(97)80046-9 9468793

[B60] Rahnama M. Novikova O. Starnes J. H. Zhang S. Chen L. Farman M. L. (2020). Transposon-mediated telomere destabilization: a driver of genome evolution in the blast fungus. *Nucleic Acids Res.* 48 7197–7217. 32558886 10.1093/nar/gkaa287 PMC7367193

[B61] Rehmeyer C. Li W. Kusaba M. Kim Y.-S. Brown D. Staben C. (2006). Organization of chromosome ends in the rice blast fungus, *Magnaporthe oryzae*. *Nucleic Acids Res.* 34 4685–4701. 10.1093/nar/gkl588 16963777 PMC1635262

[B62] Robinson J. T. Thorvaldsdóttir H. Winckler W. Guttman M. Lander E. S. Getz G. (2011). Integrative genomics viewer. *Nat. Biotechnol.* 29 24–26. 10.1038/nbt.1754 21221095 PMC3346182

[B63] Robinson N. P. Burman N. Melville S. E. Barry J. D. (1999). Predominance of duplicative *VSG* gene conversion in antigenic variation in African trypanosomes. *Mol. Cell. Biol.* 19 5839–5846. 10.1128/mcb.19.9.5839 10454531 PMC84433

[B64] Sandell L. L. Zakian V. A. (1993). Loss of a yeast telomere: arrest, recovery, and chromosome loss. *Cell* 75 729–739. 10.1016/0092-8674(93)90493-a 8242745

[B65] Starnes J. H. Thornbury D. W. Novikova O. S. Rehmeyer C. J. Farman M. L. (2012). Telomere-targeted retrotransposons in the rice blast fungus *Magnaporthe oryzae*: agents of telomere instability. *Genetics* 191 389–406. 10.1534/genetics.111.137950 22446319 PMC3374306

[B66] Thorvaldsdottir H. Robinson J. T. Mesirov J. P. (2013). Integrative genomics viewer (IGV): high-performance genomics data visualization and exploration. *Brief. Bioinform.* 14 178–192. 10.1093/bib/bbs017 22517427 PMC3603213

[B67] Watson J. M. Shippen D. E. (2007). Telomere rapid deletion regulates telomere length in *Arabidopsis thaliana*. *Mol. Cell. Biol.* 27 1706–1715. 10.1128/mcb.02059-06 17189431 PMC1820464

[B68] Wyatt N. A. Richards J. K. Brueggeman R. S. Friesen T. L. (2020). A comparative genomic analysis of the barley pathogen *Pyrenophora teres f. teres* identifies subtelomeric regions as drivers of virulence. *Mol. Plant Microbe Interact.* 33 173–188. 10.1094/mpmi-05-19-0128-r 31502507

[B69] Yu Y. Liu J. Liu X. Zhang Y. Magner E. Lehnert E. (2018). SeqOthello: querying RNA-seq experiments at scale. *Genome Biol.* 19:167. 10.1186/s13059-018-1535-9 PMC6194578 30340508

[B70] Yue J.-X. Li J. Aigrain L. Hallin J. Persson K. Oliver K. (2017). Contrasting evolutionary genome dynamics between domesticated and wild yeasts. *Nat. Genet.* 49 913–924. 10.1038/ng.3847 28416820 PMC5446901

[B71] Zakian V. A. (1989). Structure and function of telomeres. *Ann. Rev. Genet.* 23 579–604. 10.1146/annurev.ge.23.120189.003051 2694944

[B72] Zerbino D. R. Birney E. (2008). Velvet: algorithms for de novo short read assembly using de Bruijn graphs. *Genome Res.* 18 821–829. 10.1101/gr.074492.107 18349386 PMC2336801

[B73] Zlotorynski E. (2019). Telomere crisis activates autophagic death. *Nat. Rev. Mol. Cell Biol.* 20:133. 10.1038/s41580-019-0105-7 30700812

