# Supplementary material for: Telomere Roles in Fungal Genome Evolution and Adaptation
Source: Front Genet. 2021 Aug 9;12:676751. doi: 10.3389/fgene.2021.676751 (PMC8381367; doi:10.3389/fgene.2021.676751)
Supplement: Supplementary file 1 [file Presentation_1.pdf]

Supplementary Figures

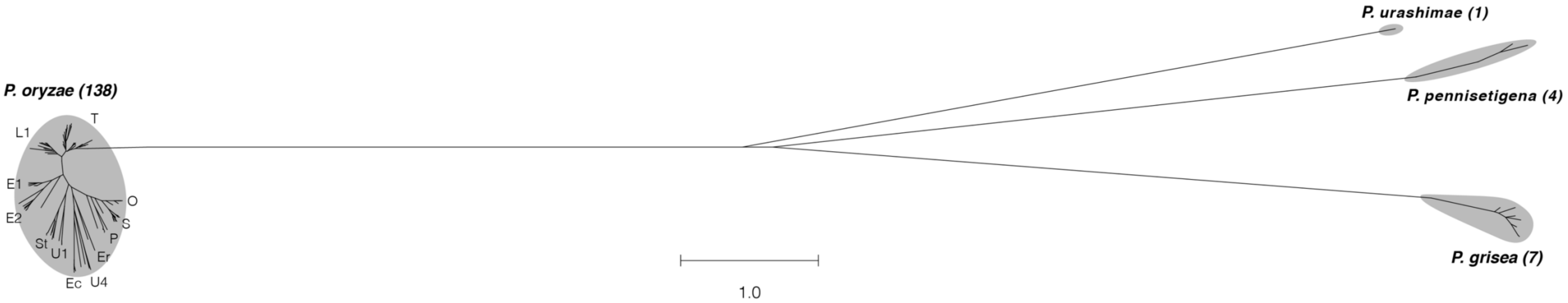

Supplementary Figure 1. Neighbor joining tree showing genetic relationships between *Pyricularia* species based on whole genome SNP analysis. Branch lengths correspond to pairwise distances between isolates (% nucleotide divergence). Gray "clouds" are draw to enclose all isolates within a given species. The numbers of isolates per species are shown in parentheses). Select host-specialized lineages of *P. oryzae* are labeled for comparison with Figure 1B (E = *Eleusine*; Ec = *Echinochloa*; Er = *Eragrostis*; L = *Lolium*; O = *Oryza*; P = *Panicum*; S = *Setaria*; St = *Stenotaphrum*; T = *Triticum* and U = *Urochloa*.

2539ΔT2

AAAAAAAAAACATTATTAAAAGTTAAAAAAGTTAGGGTTAGGGTTAGGG**TTTTCTACCCTATTTGTACGAC**

TEL12 (1654)MoTeR2(TEL10)->>

2539ΔT4

AAAAAAAAAACATTATTAAAAGTTAAAAAAGTTAGGGTTAGGGTTAGGGTTAGGGTTAGGG**TTTTAATTCGCGCTTTATT**

TEL12 <<-MoTeR1(TEL6)--

2539ΔT6

AAAAAAAAAACATTATTAAAAGTTAAAAAAGTTAGGGTTAGGGTTAGGGT**CAGGACCAATACCATGATTGA**

TEL12 TEL 3 subterminal sequence

2539ΔT10-1

AAAAAAAAAACATTATTAAAAGTTAAAAAAGTTAGGGTTAGGGTTAGGGTTAGGGTTAGGGTTAGGGTT**GAGAGCAACCAAGTGTTTAT**

TEL12 TEL11 subterminal sequence

2539ΔT10-2

AAAAAAAAAACATTATTAAAAGTTAAAAAAGTTAGGGTTAGGGTTAG**AGCGGAGCGGCAGGCCGTCTACCAA**

TEL12 TEL12 subterminal sequence

Supplementary Figure 2. Sequences of repaired TEL12 ends in four TERT KO (ΔT) lines of strain 2539. Each sequence represents the right end of chromosome 6 with the native telomere repeats underlined. Sequences added on to the resected telomeres are shown in bold, with their origins listed underneath. Underlined bold sequences represent regions of microhomology between the telomere vestige and the "captured" repair sequences. Note that two distinct repair events were recovered from 2539ΔT10.

CCCTAACCCTAACCCTAACCCTAACCCTAACCCTAACCCTAACCCTAACCCTAACCCTAACCCTACCCTAACCCTAACCCTAACCCTAA
CCCTAACCCTAACCCTAACCCTAACCCTAACCCTAACCCTAACCCTAACCCTAGCCACGTGACCAGCCGGCACGACTTTCCTCCGAGCG
GGATAAAGTTCCTCCGTAGGCGCAAGCCAAGAGGTGGCTGGGTATTACCCCGCTGTGATAACAGGGGAGGGATGCAAGTTAGCGCCGAC
CTTTGTGACGAACCATTCCCACGGAAACCAATTGTTTGGCGTCAAACACTGGCCCGTGCGCGATAATTCGTCTACTTAAAGGCTGCGTT
TTCTTCGAATCAATGCTCACAACAGCCCTGATGAGCCACAACACAGGCGAAACGTTGAAGTTCTTCCCCACGGAGACCAGCGTCGGCAA
AGTGTTCTAGTTCATCTTTTCTGCAACTTGTCTTCACCCCATCTTCAGTTACCTAACCCTAACCTTGAGACAAG**ATGGCTTCTAAGTCA**
ATTCGGTCTGTGGACATCATCACTACCAGCAACCCCATCGCTGTGGCAACTGCTAGGGTCACCACGATCGGGGAATTGATCTCCACCAT
CCCACAAACATGGCGCCCCGCGCTGGCACCCTACCTTCAGAACGTCTACAGGCTTGCCAGCAAACTATGCACAGTCAGCAACACTGTCG
CACAATACGAGCGTCATAAATTGACAGGTTCCTTCCCTCCCTTCGTTATTAACTCAATCAAGGAACCAAAAGTCCAGTTCTCGAAAGAA
TTCCGAGGCACGGCTTCAGGCAAGTCTGCAGAAGACAACCTGAAAACCGGTACCATTCAAGCACGCAAGAACCTCCTCAAAGATGCGAT
CGCTTACAAACAAGAGGAACTCTCAGAACTCCAGATCGCGATCAAGTTTGACGCCGAGCAGTGGACCACACTCATGACGCCGGTCATGG
AGAGAGTTGCCCAAACCCTGGGCGCCACTATTAAGTGGTCAGAGAAAGGCGCCCCCATCTGGACTGGGGACACCGATTGCAAGGAACTC
CGAGAAGAATCGAAGAGTTTGCTTGACAACGGTTCTGTTTTACATTACCGGGCTGTCGCACTCGCTAGATCTCTCGCAGACAAGTCACT
TGTTGAGAAGACAAAGTTACTGTCGCTCAAAAAGTCGACTGATGTCGAAATGCGCGATGTCGACACCGAGAAGACTACTCGGGAAGTCG
TCCGTGATGAGCTTGACGCTAAGTTCGCTCAATTCCAACAAAAGATTGCTGGAATGATTGGTACGTCGTCACTTGGCAAACGCCGTGGC
TTGGTACTAACTTCCGACAGGCAACCAAAGAAAGTCCGGCCCGAAAGGGAACGGCCAGAACAAGCCCCAGTCAAAGGGCAAGATTGTCA
AGAAGACATCTCCGAAGAAGAAGCAAGGAAGACGATGACGACTTCAGCTTTTCTTTCTCAGTGTTCTAGAAGTTTTCGCAGTTACGACG
CGGAAACTTACCCCGCAGTGTATGCAAGTTTGTCTTCATCATGTAGAATTAAGATTGCAGTCTCTCTTATGAGAGAATGGGAACTCGGA
ACGTGGCACACGGCCGAACCCGGAGTATTTCAACATGCCAGTGTCGACGTGCCAAGCGACATTGGTTACATGTTAGCTGTCAATCATAA
ATTCATTCTTCATCAAGCCCCCTCACTGCATGACATAGACGAAGCCAAGTATAGGCTACGTCGCTCGGTTCGCAACCGATGGTTCTTCC
GAGAAAAGACGAGTCGGGACTTTATCCCAAAGTTCCACATACCTAACAACAAGTGGCAACCGCCCCCAGCATCAAACTGGATTGAACAG
GGTCTAGATGCAGCCGAGAAAGTGATCGATGCGCAATGCGCTCGGGCCCTTGCGAGCTCCGCTGTACGACCAACTTCCAGGCCCTTCAA
AGCCTGGAAAACTGTTCAAGATTACCTTGCCCAGCATGAGCTCTTGGCAAAGCTGACGGACAAGAATCTTGGCCTTGCGGTTATGCCAC
TCCAATGGTATGATACAGAGATTCTCATGATGCTTGCTGATGGCAACACCTATGAAAGGGTTGCGTCAGTACCTACATCAGGACTCGTC
AAGGAAATGATCAGTCTCCTTCCCCAATGGAGACTTCCTAAACAACTGGACAAGTATGTAAAGACACCGAAAAAGCTGGAGCTCCCGGA
ATTTCATTGTATACCAAAGGTACATAAAAATCCGTGGACGCTTAGACCCATCGTGCCGTCACACTCATGGGTCACAACATCAGTGTCCG
AAGTACTAGATCATCTCCTTCAGCCCCTTCTCGAGCACTTCCCCTGGGTTGTCGCATCTTCAAAAGATGTCATCCTACAAATCGAGAAA
GTGCGCTCTACAAATGTTAAGCCTGTGGTGATTGCAACAGGAGATGTGACCTCCTTCTACACAAACATCCCACCCAAGGACTGCGCAAA
AGTAATTGCCGGTGCCTGGAAACTTTACATGAGTGGATCGACCATTCCAGCATCTGCCATTCGCAAAATGGTAGATTTTGTTATGGTCA
ACAACTTCTTTCAGTACCGCGGTCAAACGTTCCGACAGAAGATGGGTCTTGCCATGGGCACGTCGTGCGCCCCAGTGCTTGCCAACATC
TATGCTGCTTACTTTGAACGAAAGGCCAAGATTGTCAATCAAGAAGGAGTTTTACTCTACGTTCGATACATTGATGACATTCTCTGCAT
TTTACAGGGTACTAAAGAAGAACAACGTAAATTCGTTCAGAGATTCCAATTGGGAAATCTGACAGTACGTTGGGACAGTTCAGAAGAAC
GGAATGAGTTTCTCGACATTGAGTTGGTCCGAGTAGCTCTTGAGGGCAGCCTCAGAGTCTGTCATACAAGACTCTTCCGGAAACCTATG
AACCGTTTCCTCTACATTCCCTGGTCTTCAGCTCACCCCTTGCACGTCAAGAAAGGCTTTGTCAAAGCCGAGCTTACAAGATTTGCCAT

ACTCTGCTCCAAATACGAGTTCTTTGCAGAAGCCCGTATGGAATTTTATGGCAATCTCCGTCGTAGGGGATACCCGACGGAGACACTGA
GAGAGTGGTTTCAACAGATTCAATATGACGACAGAGCCCGTATCTTACTTCCCAAACAGAAGAAAGACACTGGAGCACCTCTTATGTTG
TCAGGCCACTATAACCCAGTGTGGGATTATGTAGACGTCAAGGAGGTACTCGATGCAGCACGAATGCTGTGGACAAAGGAAGAGCTGCC
GGAGTCCTTAGAACAGCCGCTGATTCGAAGCCTTGGTCGAACCACAAGCCTTTTCGACTTGTTATCGACATGGAACAAGACACTTCTGC
TCCTTCCTTCGGAAGGGGGGCCGAGTTAGTAGGAACGAAAAGCTAGCAGGCAACGTACCCGATGGGGGTGGGTCTCCCCGCTGCAAAGT
GGCCCATAAGAGAGACCTTGTCGGGTGACCCTTGACGGTACGTAGAGACGTGGGACCATCTCTGCAAATGCCACCCGAGCCAGACTAGT
ACAAACGCTCGTGTCCATGCCTCTCACCATGGGATTAGCCCTACTTGTAGATCACCGAAGAAGCCGACCAGAGACAGCCCTGCGGGGTA
CGGAATGGCAATCCGAGTTAAATGTCAGCCGGATGTTATTAGTCTTCTGACTCGGGGCCAAGTTCTTGGGCTATGATTGCCGGCGATGC
ATGCGTCCTCTCCGTCGCAGGAAGATTTTCAGATTTCCACTTGACAAAACAGCATTTTTGGTTAGGTCTTTGACAGGCCGGATCGAACC
CGGACCACTAACCACTAACCCTAACCCTAGCCACGTGACCAGCCGGCACGACTTTCCTCCGAGCGGGATAAAGTTCCTCCGTAGGCGCA
AGCCAAGAGGTGGCTGGGTATTACCCCGCTGTGATAACAGGGGAGGGGATGCAAGTTAGCGCCGACCTTTGTGACGAACCATTCCCACG
GAAACCAATTGTTTGGCGTCAAACACTGGCCCGTGCGCGATAATTCGTCTACTTAAAGGCTGCGTTTTCTTCGAATCAATGCTCACAAC
AGCCCTGATGAGCCACAACACAGGCGAAACGTTGAAGTTCTTCCCCACGGAGACCAGCGTCGGCAAAGTGTTCTAGTTCATCTTTTCTG
CAACTTGTCTTCACCCCATCTTCAGTTACCTAACCCTAACC

Supplementary Figure 3. **Sequence of the subtelomeric tandem repeat in the foxtail pathogens showing an LTR retrotransposon-like structure.** Telomere motifs are shown in red – the actual telomere is shown in italics, and the long terminal repeat-like structures are underlined. The reverse transcriptase open reading frame is highlighted in bold.
