# Supplementary material for: Telomere Roles in Fungal Genome Evolution and Adaptation
Source: Front Genet. 2021 Aug 9;12:676751. doi: 10.3389/fgene.2021.676751 (PMC8381367; doi:10.3389/fgene.2021.676751)
Supplement: Supplementary file 7 [file Data_Sheet_6.docx]

**Supplementary Table 1:** Information on sequence datasets used in this study

| **Strain** | **Alt. names** | **Host of isolation** | **Country** | **Locality** | **Year** | **Reference** | **Phylogenet. lineage^A^** | **NCBI accession no.** |
| --- | --- | --- | --- | --- | --- | --- | --- | --- |
| Br58 |  | *Avena sativa* (Oat) | Brazil | PR | 1990 | Yoshida, et al. 2016 | L1 | SAMD00051172 |
| As345 | 12.0.345 | *Avena sativa* (Oat) | Brazil | MS | 2012 | Castroagudín, et al. 2016 | L1 | SAMN07829574 |
| P28 | P-0028 | *Bromus tectorum (cheat grass)* | Paraguay | IT | 2014 | Pieck, et al. 2017 | L1 | SAMN05864041 |
| P29 | P-0029 | *B. tectorum* | Paraguay | IT | 2014 | Pieck, et al. 2017 | T | SAMN05898532 |
| Ce642i | 12.0.642i | *Cenchrus echinatus* (Buffel grass) | Brazil | PR | 2012 | Castroagudín, et al. 2016 | T | SAMN07829578 |
| Ce88454 |  | *C. echinatus* | Philippines | Bulacan | 1988 | Borromeo, et al. 1993 | C1 | SAMN19488801 |
| MG07 |  | *C. ciliaris* | India | Bangalore, KA |  | Shirke, et al. 2016 | C2 | SAMN04217096 |
| Cd88215 |  | *Cynodon dactylon* (Bermuda grass) | Philippines | Cabanatuan, NE | 1988 | Borromeo, et al. 1993 | C1 | SAMN14167123 |
| Cd88217 |  | *C. dactylon* | Philippines | Cabanatuan, NE | 1988 | Borromeo, et al. 1993 | C1 | SAMN14167123 |
| CpJA159 |  | *Cynodon plectostachyus* (Bermudagrass) | Brazil | MG | 2018 | Present study | C1 | SAMN19488802 |
| BR29 |  | *Digitaria sanguinalis* (Hairy crabgrass) | Brazil | Goias | 1989 | Gladieux, et al. 2018 | Pg | SAMEA3108252 |
| Dig41 |  | *D. sanguinalis* | Japan | Hyogo |  | Yoshida, et al. 2016 | Pg | SAMD00051174 |
| Ds363 |  | *D. sanguinalis* | Brazil | PR | 2012/2013 | Castroagudín, et al. 2017 | Ec | SAMN19488803 |
| Ds555i | 12.0.555i | *D. sanguinalis* | Brazil | PR | 2012 | Castroagudín, et al. 2016 | L1 | SAMN07829577 |
| DsLIZ |  | *D. sanguinalis* | USA | KY | 2000 | Gladieux, et al. 2018 | Pg | SAMN08009550 |
| P26 | P-0026 | *D. sanguinalis* | Paraguay | Canindeyú |  | Present study | Pg | SAMN19488804 |
| NI907 |  | *D. sanguinalis* | Japan | Tochigi | 1974 | Ikeda et al. 2013 | Pg | SAMN10496230 |
| P30 | P-0030 | *D. sanguinalis* | Paraguay | Itapúa |  | Present study | Pg | SAMN19488805 |
| U167 |  | *D. sanguinalis* | Uruguay | Lavelleja | 2010 | Yasuhara-Bell, et al. 2018 | Pg | SAMN19488806 |
| VO107 |  | *D. sanguinalis* | USA | TX | 1981 | Gladieux, et al. 2018 | Pg | SAMN08009577 |
| U170 |  | *Echinochloa sp.* (Jungle rice) | Uruguay | Treinta y Tres | 2010 | Yasuhara-Bell, et al. 2018 | Pg | SAMN19488807 |
| Ec88443 |  | *Echinochloa colona* (Jungle rice) | Philippines | Los Baños, LB | 1988 | Borromeo, et al. 1993 | Ec | SAMN19488808 |
| Ecrus326 | 12.0.326 | *Echinochloa crusgalii* | Brazil | MS | 2012 | Castroagudín, et al. 2016 | L1 | SAMN07829573 |
| G22 |  | *Eleusine coracana* (finger millet) | Japan | Unknown | 1976 | Gladieux, et al. 2018 | E2 | SAMN08009554 |
| JP29 |  | *E. coracana* | Japan | Unknown | 1991 | Tredway, et al., 2003 | Er | SAMN19488809 |
| PH42 |  | *E. coracana* | Philippines | Unknown | 1983 | Farman, et al. 2017 | E1 | SAMN08009570 |
| Z2-1 |  | *E. coracana* | Japan | Kagawa | 1977 | Yoshida, et al. 2016 | E2 | SAMD00051173 |
| MG03 |  | *E. coracana* | India | Bangalore, KA | 2013 | Shirke, et al. 2016 | E2 | SAMN04216994 |
| MG04 |  | *E. coracana* | India | Bangalore, KA | 2012 | Shirke, et al. 2016 | E2 | SAMN04216996 |
| MG12 |  | *E. coracana* | India | Bangalore, KA | 2013 | Shirke, et al. 2016 | E2 | SAMN04217237 |
| B51 |  | *Eleusine indica* (goose grass) | Bolivia | Quirusillas, SC | 2012 | Farman, et al. 2017 | E1 | SAMN08009542 |
| Br62 |  | *E. indica* | Brazil | Unknown | 1991 | Islam, et al. 2016 | E1 | SAMEA4029901 |
| CD156 |  | *E. indica* | Ivory Coast | Ferkessedougou, SV | 1989 | Chiapello, et al. 2015 | E1 | SAMEA4708261 |
| Ei534i | 12.0.534i | *E. indica* | E. indica | PR | 2012 | Castroagudín, et al. 2016 | T | SAMN07829576 |
| Ei8303 | EiA8303 | *E. indica* | Philippines | Los Baños, LB | 1984 | Borromeo, et al. 1993 | E1 | SAMN19488810 |
| Ei88365 |  | *E. indica* | Philippines | Santo Tomas, BTG | 1988 | Borromeo, et al. 1993 | E1 | SAMN19488811 |
| Ei8927 |  | *E. indica* | Philippines | BUDA | 1989 | Borromeo, et al. 1993 | E1 | SAMN19488812 |
| Ei9064 |  | *E. indica* | China | FJ | 1996 | Borromeo, et al. 1993 | E1 | SAMN19488813 |
| Ei9411 |  | *E. indica* | China | FJ | 1990 | Borromeo, et al. 1993 | E2 | SAMN04318447 |
| EiJA22 |  | *E. indica* | Brazil | Patos de Minas, MG | 2018 | Present study | T | SAMN19488814 |
| EiJA178 |  | *E. indica* | Brazil | Viçosa, MG | 2018 | Present study | E3 | SAMN19488815 |
| EiJA56 |  | *E. indica* | Brazil | Viçosa, MG | 2018 | Present study | E3 | SAMN19488816 |
| MZ5-1-6 |  | *E. indica* | Japan | Unknown |  | Inoue, et al. 2017 | E2 | SAMD00069327 |
| U229 |  | *E. indica* | Uruguay | Valle Alto, TT | 2017 | Yasuhara-Bell, et al. 2018 | E1 | SAMN19488817 |
| U231 |  | *E. indica* | Uruguay | Valle Alto, TT | 2017 | Yasuhara-Bell, et al. 2018 | E1 | SAMN19488818 |
| U169-v1 | U169 | *Eleusine spp.* | Uruguay | Río Branco, CL | 2010 | Yasuhara-Bell, et al. 2018 | E1 | SAMN19488819 |
| AR4 |  | *Eragrostis curvula* (weeping lovegrass) | Japan | Unknown |  | Chao & Ellingboe 1991 | Er | SAMN19488820 |
| Er88271 |  | *Eragrostis sp.* | Phillipines | Los Baños, LB | 1988 | Borromeo, et al. 1993 | Pg | SAMN19488821 |
| P25 | P-0025 | *Eragrostis sp.* | Paraguay | Canindeyú | *Eragrostis sp.* | Yasuhara-Bell, et al. 2018 | L1 | SAMN19488822 |
| G17 |  | *E. curvula* | Japan | Unknown | 1976 | Islam, et al. 2016 | Er | SAMN08009553 |
| EtKY19-1 |  | *Eragrostis tef* (Teff) | USA | KY | 2019 | Rahnama, et al. 2020 | Er | SAMN13964779 |
| pg1213-2 |  | *Festuca arundinacea* (Tall fescue) | USA | GA | 1999/2000 | Yasuhara-Bell, et al. 2018 | L2 | SAMN14603777 |
| pg1213-22 |  | *F. arundinacea* | USA | GA | 1999/2000 | Pieck, et al. 2017 | L1 | SAMN08009569 |
| TF05-1 |  | *F. arundinacea* | USA | Lexington, KY | 2005 | Gladieux, et al. 2018 | L1 | SAMN08009576 |
| TF15-1 |  | *F. arundinacea* | USA | Lexington, KY | 2015 | Present study | L1 | SAMN14144147 |
| FPH-2015-44 |  | *Hakonechloa macra* (Hakone grass) | USA | OH | 2015 | Yasuhara-Bell, et al. 2018 | H | SAMN14144145 |
| BTBa-B1 |  | *Hordeum vulgare* (Barley) | Bangladesh | Gazipur | 2016 | Soanes, et al. 2017 | T | SAMEA104190806 |
| BTBa-B2 |  | *H. vulgare* | Bangladesh | Gazipur | 2016 | Soanes, et al. 2017 | T | SAMEA104190807 |
| TH0012-rn | TH0012, TH12 | *H. vulgare* | Thailand | Unknown | Unknown | Islam, et al. 2016 | O | SAMEA3231789 |
| TH0016 | TH16 | *H. vulgare* | Thailand | Unknown | Unknown | Islam, et al. 2016 | O | SAMEA3232033 |
| Lh8401 | LhA8401 | *Leersia hexandra* (Southern cutgrass) | Philippines | Los Baños, LB | 1984 | Borromeo, et al. 1993 | Le | SAMN19488823 |
| Lh88405-2 | Lh88405 | *L. hexandra* | Philippines | Los Baños, LB | 1988 | Borromeo, et al. 1993 | Le | SAMN14167125 |
| Lh8844 |  | *L. hexandra* | Philippines | Cabanatuan, NE | 1988 | Borromeo, et al. 1993 | Le | SAMN19488824 |
| Lc8401 | LcA8401 | *Leptochloa chinensis* (Red sprangletop) | Philippines | Los Baños, LB | 1984 | Borromeo, et al. 1993 | Ec | SAMN14144146 |
| ATCC64557 | U49 | *Lolium multiflorum* (Festuca perennis*)* | USA | MS | 1972 | Tosa, et al. 2007 | L1 | SAMN19488825 |
| PL2-1 |  | *L. multiflorum* | USA | Pulaski Co., KY | 2002 | Inoue, et al. 2017 | L1 | SAMN08009571 |
| PL3-1 |  | *L. multiflorum* | USA | Pulaski Co., KY | 2002 | Farman, et al. 2017 | L1 | SAMN08009572 |
| Po221 |  | *L. multiflorum* | Uruguay | Cerro Largo | 2015 | Present study | L1 | SAMN14153273 |
| U234 |  | *L. multiflorum* | Uruguay | 18 de Julio, RO | 2017 | Yasuhara-Bell, et al. 2018 | L1 | SAMN19488826 |
| U235 |  | *L. multiflorum* | Uruguay | 18 de Julio, Rocha | 2017 | Yasuhara-Bell, et al. 2018 | L1 | SAMN19488827 |
| U237 |  | *L. multiflorum* | Uruguay | Treinte y Tres | 2017 | Yasuhara-Bell, et al. 2018 | Ec | SAMN19488828 |
| CHRF |  | *Lolium perenne* (perennial ryegrass) | USA | Siler Springs, MD | 1996 | Inoue, et al. 2017 | L1 | SAMN08009548 |
| CHW |  | *L. perenne* | USA | Severna Park, MD | 1996 | Inoue, et al. 2017 | L1 | SAMN08009549 |
| FH |  | *L. perenne* | USA | Hagerstown, MD | 1997 | Pieck, et al. 2017 | L1 | SAMN08009551 |
| GG11 |  | *L. perenne* | USA | Lexington, KY | 1997 | Farman, et al. 2017 | L1 | SAMN08009555 |
| HO |  | *L. perenne* | USA | Richmond, PA | 1996 | Gladieux et al. 2018 | L1 | SAMN08009558 |
| LpKY97 | LpKY97-1 | *L. perenne* | USA | Lexington, KY | 1997 | Farman, et al. 2017 | L1 | SAMN08009564 |
| PgKY | PgKY4OV2.1 | *L. perenne* | USA | Lexington, KY | 2000 | Islam, et al. 2016 | L1 | SAMEA4029903 |
| PgPA | PgPA18C-02, PGPA | *L. perenne* | USA | PA | 1998 | Islam, et al. 2016 | L1 | SAMEA4029904 |
| TP2 |  | *L. perenne* | Japan | Tochigi | 1997 | Inoue, et al. 2017 | L1 | SAMN14151737 |
| Wk3-1 |  | *L. perenne* | Japan | Yamaguchi | 1996 | Tosa et al., 2004 | L3 | SAMN14603776 |
| U168 |  | *Luziola peruvianum* (watergrass) | Uruguay | Río Branco, CL | 2010 | Yasuhara-Bell, et al. 2018 | Lu | SAMN14603775 |
| U171 |  | *L. peruvianum* | Uruguay | Zapata, TT | 2010 | Yasuhara-Bell, et al. 2018 | Lu | SAMN19488829 |
| 87-120 |  | *Oryza sativa* (rice) | Unknown | Unknown | Unknown | Gladieux, et al. 2018 | O | SAMN08377452 |
| FR13 |  | *O. sativa* | France | Unknown | 1990 | Faivre-Rampant, et al. 2008 | O | SAMEA4708258 |
| Guy11 |  | *O. sativa* | French Guyana | Unknown | 1988 | Islam, et al. 2016 | O | SAMN06050151 |
| IA1 | ARB114 | *O. sativa* | USA | AR | 2009 | Pieck, et al. 2017 | O | SAMN08009559 |
| IB33 |  | *O. sativa* | USA | AR | unknown | Present study | O | SAMN08009560 |
| IB49 | ZN61 | *O. sativa* | USA | AR | 1992 | Pieck, et al. 2017 | O | SAMN08009561 |
| IC17 | ZN57 | *O. sativa* | USA | AR | 1992 | Pieck, et al. 2017 | O | SAMN08009562 |
| IE1K | TM2 | *O. sativa* | USA | AR | 2003 | Pieck, et al. 2017 | O | SAMN08009563 |
| INA168 |  | *O. sativa* | Japan | Aichi | 1958 | Inoue, et al. 2017 | O | SAMD00051169 |
| Ken53-33 |  | *O. sativa* | Japan | Aichi | 1953 | Yoshida, et al. 2016 | O | SAMD00051177 |
| MBSD02 | RMg-Dl | *O. sativa* | India | BR | 2016 | Kumar, et al. 2017 | O | SAMN05425585 |
| ML33 |  | *O. sativa* | Mali | Unknown | 1995 | Gladieux, et al. 2018 | O | SAMN08009565 |
| P-2 |  | *O. sativa* | Japan | Aichi | 1948 | Yoshida, et al. 2016 | O | SAMD00051176 |
| P131 |  | *O. sativa* | Japan | Unknown | Unknown | Farman, et al. 2017 | O | SAMN02981399 |
| PH0014-rn |  | *O. sativa* | Philippines | Unknown | Unknown | Islam, et al. 2016 | O | SAMN19488830 |
| Rmg_DI |  | *O. sativa* | India | Bihar | 2012 | Present study | O | SAMN19488831 |
| SSID116 |  | *O. sativa* | USA | Unknown | 1997 | Yasuhara-Bell, et al. 2018 | O | SAMN19488832 |
| TH3 |  | *O. sativa* | Thailand | Unknown | ND | Yoshida, et al. 2016 | O | SAMD00051175 |
| U75 |  | *O. sativa* | Uruguay | Treinte y Tres | 2005 | Yasuhara-Bell, et al. 2018 | O | SAMN19488833 |
| U107 |  | *O. sativa* | Uruguay | Treinte y Tres | 2009 | Present study | O | SAMN19488834 |
| U198 |  | *O. sativa* | Uruguay | Tacuarembó | 2014 | Present study | O | SAMN19488835 |
| Y34 |  | *O. sativa* | China | YN | 1982 | Xue, et al. 2012 | O | SAMN02981398 |
| BTTrp-5 |  | *Panicum repens* (Torpedograss) | Bangladesh | Gazipur | 2016 | Soanes, et al. 2017 | P | SAMEA104190823 |
| BTTrp-6 |  | *P. repens* | Bangladesh | Gazipur | 2017 | Soanes, et al. 2017 | P | SAMEA104190824 |
| Pr8202 | PrA8202 | *P. repens* | Philippines | Los Baños, LB | 1982 | Borromeo, et al. 1993 | P | SAMN14603774 |
| Pr88165 |  | *P. repens* | Philippines | Cabanatuan, NE | 1989 | Borromeo, et al. 1993 | P | SAMN19488836 |
| Pd88413 |  | *Paspalum distichum* (knotgrass) | Philippines | Los Baños, LB | 1988 | Borromeo, et al. 1993 | Ec | SAMN14144143 |
| ML36 |  | *Pennisetum sp.* | Mali |  |  | Present study | Pp | SAMN19488837 |
| Pm1 |  | *Pennisetum americanum* | USA | GA | 1990 | Gladieux, et al. 2018 | Pp | SAMN08377453 |
| PtKY18-1 |  | *Poa trivialis* (Rough bluegrass) | USA | Lexington, KY | 2018 | Present study | L1 | SAMN19488838 |
| RrJA49 |  | *Romulea rosea* (Guildford grass) | Brazil | MG | 2018 | Present study | U4 | SAMN19488839 |
| BP1-FLA |  | *Setaria faberi* (Japanese bristlegrass) | USA | FL | 2018 | Present study | S | SAMN19488840 |
| GFSI1-7-2 |  | *Setaria italica* (foxtail millet) | Japan | Gifu | 1977 | Yoshida, et al. 2016 | S | SAMD00051170 |
| MG05 |  | *S. italica* | India | Bangalore, KA | 2012 | Shirke, et al. 2016 | S | SAMN04217000 |
| MG08 |  | *S. italica* | India | Mandya, KA | 2012 | Shirke, et al. 2016 | S | SAMN04217082 |
| U232 |  | *S. italica* | Uruguay | Minas, LA | 2017 | Yasuhara-Bell, et al. 2018 | S | SAMN19488841 |
| US71 |  | *Setaria spp.* | USA | Lexington, KY | ND | Chiapello, et al. 2015 | S | SAMEA3373385 |
| Arcadia2 |  | *Setaria viridis* (green foxtail) | USA | Lexington, KY | 1998 | Farman, et al. 2014 | S | SAMN14167122 |
| GRF52 |  | *S. viridis* | USA | Lexington, KY | 2001 | Gladieux, et al. 2018 | S | SAMN08009556 |
| KANSV1-4-1 |  | *S. viridis* | Japan | Kanagawa | 1975 | Yoshida, et al. 2016 | S | SAMD00051178 |
| SA05-144 |  | *S. viridis* | Japan | Nagasaki | 2005 | Yoshida, et al. 2016 | S | SAMD00051180 |
| SA05-43 |  | *S. viridis* | Japan | Nagasaki | 2005 | Yoshida, et al. 2016 | S | SAMD00051179 |
| Sv9610 |  | *S. viridis* | China | ZJ | 1996 | Zhong, et al. 2016 | S | SAMN04318449 |
| Sv9623 |  | *S. viridis* | China | ZJ | 1996 | Zhong, et al. 2016 | S | SAMN04318450 |
| Pg1054 |  | *Stenotaphrum secundatum* (St. Augustinegrass) | USA | GA | 1999/2000 | Yasuhara-Bell, et al. 2018 | St | SAMN19488842 |
| Pg1204 |  | *S. secundatum* | USA | GA | 1999/2000 | Present study | St | SAMN19488843 |
| SSFL02-1 | SSFL02 | *S. secundatum* | USA | Disneyworld, FL | 2002 | Pieck, et al. 2017 | St | SAMN08009573 |
| SSFL14-3 |  | *S. secundatum* | New Smyrna, FL | New Smyrna, FL | 2014 | Gladieux, et al. 2018 | St | SAMN08009574 |
| SSTX16-11 | SSTX16-1 | *S. secundatum* | USA | TX | 2016 | Yasuhara-Bell, et al. 2018 | St | SAMN14144144 |
| STAG-MS |  | *S. secundatum* | USA | MS | 1980 | Present study | St | SAMN19488844 |
| U217 |  | *S. secundatum* | Uruguay | Treinte y Tres | 2015 | Yasuhara-Bell, et al. 2018 | St | SAMN19488845 |
| U233 |  | *S. secundatum* | Uruguay | Covidef 1, FL | 2017 | Yasuhara-Bell, et al. 2018 | St | SAMN19488846 |
| B2 |  | *Triticum aestivum* (wheat) | Bolivia | Quirusillas, SC | 2011 | Inoue, et al. 2017 | T | SAMN05580113 |
| B71 |  | *T. aestivum* | Bolivia | Quirusillas, SC | 2012 | Inoue, et al. 2017 | T | SAMN04942725 |
| BdBar | P161 | *T. aestivum* | Bangladesh | Barisal | 2016 | Inoue, et al. 2017 | T | SAMN04940126 |
| BdJes | P162 | *T. aestivum* | Bangladesh | Jessore | 2016 | Inoue, et al. 2017 | T | SAMN04942531 |
| BdKUS |  | *T. aestivum* | Bangladesh | Kushtia | 2016 | Inoue, et al. 2017 | T | SAMN14144137 |
| BdMag |  | *T. aestivum* | Bangladesh | Magura | 2016 | Inoue, et al. 2017 | T | SAMN14144138 |
| BdMeh | P163 | *T. aestivum* | Bangladesh | Mehepur | 2016 | Inoue, et al. 2017 | T | SAMN04942534 |
| BR116 | Br116.5 | *T. aestivum* | Brazil | PR | 1992 | Inoue, et al. 2017 | T | SAMN14144139 |
| BR118 |  | *T. aestivum* | Brazil | PR | 1992 | Inoue, et al. 2017 | T | SAMN14144140 |
| Br130 |  | *T. aestivum* | Brazil | MS | 1990 | Farman, et al. 2017 | T | SAMN08009547 |
| BR32 | BR0032 | *T. aestivum* | Brazil | Unknown | 1991 | Chiapello, et al. 2015 | T | SAMEA4708260 |
| Br48 |  | *T. aestivum* | Brazil | MS | 1990 | Yoshida, et al. 2016 | T | SAMD00084261 |
| Br7 |  | *T. aestivum* | Brazil | PR | 1990 | Urashima, et al. 1999 | T | SAMN08009545 |
| Br80 |  | *T. aestivum* | Brazil | Unknown | 1991 | Farman, et al. 2017 | T | SAMN08009546 |
| BR81 |  | *T. aestivum* | Brazil | Unknown | 1991 | Couch, et al. 2005 | T | SAMN14144142 |
| BTGP-1b |  | *T. aestivum* | Bangladesh | Mehepur | 2017 | Soanes, et al. 2017 | T | SAMN19488847 |
| BTGP-6e |  | *T. aestivum* | Bangladesh | Mehepur | 2017 | Soanes, et al. 2017 | T | SAMN19488848 |
| BTJP4-1 |  | *T. aestivum* | Bangladesh | Mehepur | 2017 | Soanes, et al. 2017 | T | SAMEA4708257 |
| PY0925 |  | *T. aestivum* | Brazil | Predizes, SP | 2009 | Islam, et al. 2016 | T | SAMEA4029894 |
| Py221 | Py22.1 | *T. aestivum* | Brazil | PR | 2007 | Pieck, et al. 2017 | T | SAMN05725179 |
| PY36 | PY36.1 | *T. aestivum* | Brazil | Brasilia, DF | 2007 | Yasuhara-Bell, et al. 2018 | T | SAMEA4029897 |
| PY5003 |  | *T. aestivum* | Brazil | Londrina, PR | 2005 | Yasuhara-Bell, et al. 2018 | T | SAMEA4029888 |
| PY5010 |  | *T. aestivum* | Brazil | Londrina, PR | 2005 | Islam, et al. 2016 | T | SAMEA4029898 |
| Py5020 |  | *T. aestivum* | Brazil | Londrina, PR | 2005 | Pieck, et al. 2017 | T | SAMN05762829 |
| PY5033 | PY05033 | *T. aestivum* | Brazil | Londrina, PR | 2005 | Islam, et al. 2016 | T | SAMEA4029889 |
| PY6017 | PY06017 | *T. aestivum* | Brazil | Coromandel, MG | 2006 | Islam, et al. 2016 | T | SAMEA4029890 |
| PY6025 |  | *T. aestivum* | Brazil | MG | 2006 | Inoue, et al. 2017 | T | SAMEA4029891 |
| PY6045 |  | *T. aestivum* | Brazil | GO | 2006 | Inoue, et al. 2017 | T | SAMEA4029900 |
| PY86 | PY86.1 | *T. aestivum* | Brazil | PR | 2008 | Inoue, et al. 2017 | L1 | SAMEA4029893 |
| T12-8 |  | *T. aestivum* | Brazil | Floresta, PR | 1988 | Present study | T | SAMN19488849 |
| T13-3 |  | *T. aestivum* | Brazil | Floresta, PR | 1988 | Present study | T | SAMN19488850 |
| T1-1 |  | *T. aestivum* | Brazil | Camp Grande, MS | 1988 | Present study | T | SAMN19488851 |
| T2-1 | T-0002 | *T. aestivum* | Brazil | Londrina, PR | 1987 | Present study | T | SAMN19488852 |
| T21-1 |  | *T. aestivum* | Brazil | Floresta, PR | 1988 | Present study | T | SAMN19488853 |
| T25 |  | *T. aestivum* | Brazil | São Jorge do Ivaí, PR | 1988 | Present study | T | SAMN08009575 |
| T3-1 |  | *T. aestivum* | Brazil | Vicentinópolis, GO | 1986 | Present study | T | SAMN19488854 |
| T30-2 |  | *T. aestivum* | Brazil | PR | 1989 | Present study | T | SAMN19488855 |
| T37-2 |  | *T. aestivum* | Brazil | PR | 1989 | Present study | T | SAMN19488856 |
| T4-2 |  | *T. aestivum* | Brazil | Floresta, PR | 1988 | Present study | T | SAMN19488857 |
| T42-2 |  | *T. aestivum* | Brazil | PR | 1989 | Present study | T | SAMN19488858 |
| T46-2 |  | *T. aestivum* | Brazil | PR | 1989 | Present study | T | SAMN19488859 |
| T47-3 |  | *T. aestivum* | Brazil | PR | 1985 | Present study | T | SAMN19488860 |
| T5-3 |  | *T. aestivum* | Brazil | Palotina, PR | 1988 | Present study | T | SAMN19488861 |
| T50-3 | T-0050 | *T. aestivum* | Brazil | MG | 1989 | Present study | T | SAMN19488862 |
| WB032i | 12.1.032i | *T. aestivum* | Brazil | PR | 2012 | Castroagudín, et al. 2016 | T | SAMN19488863 |
| WB053i | 12.1.053i | *T. aestivum* | Brazil | SP | 2012 | Castroagudín, et al. 2016 | T | SAMN19488864 |
| WB127 | 12.1.127 | *T. aestivum* | Brazil | MA | 2012 | Castroagudín, et al. 2016 | T | SAMN19488865 |
| WB169 | 12.1.169 | *T. aestivum* | Brazil | MA | 2012 | Castroagudín, et al. 2016 | T | SAMN19488866 |
| WB205 | 12.1.205 | *T. aestivum* | Brazil | RN | 2012 | Castroagudín, et al. 2016 | T | SAMN18576983 |
| WB37 | 12.1.037 | *T. aestivum* | Brazil | GO | 2012 | Castroagudín, et al. 2016 | T | SAMN18576980 |
| WBKY11 | WBKY11-15 | *T. aestivum* | USA | Lexington, KY | 2011 | Farman, et al. 2017 | T | SAMN08009578 |
| WBSS |  | *T. aestivum* | Brazil | Unknown | Unknown | Farman, et al. 2017 | T | SAMN08009579 |
| WHTQ |  | *T. aestivum* | Brazil | Unknown | Unknown | Present study | T | SAMN08009580 |
| P3 |  | *Triticum* *durum* (Durum wheat) | Paraguay | CY | 2012 | Pieck, et al. 2017 | T | SAMN08009568 |
| Ub007i | 12.0.007i | *Urochloa brizantha* (Palisade grass) | Brazil | PR | 2012 | Castroagudín, et al. 2016 | T | SAMN07829570 |
| Ub009i | 12.0.009i | *U. brizantha* | Brazil | PR | 2012 | Castroagudín, et al. 2016 | T | SAMN07829571 |
| Ub012i | 12.0.012i | *U. brizantha* | Brazil | PR | 2012 | Castroagudín, et al. 2016 | T | SAMN07829572 |
| Ub368 | 12.0.368 | *U. brizantha* | Brazil | MS | 2012 | Castroagudín, et al. 2016 | L1 | SAMN07829575 |
| UbJA112 |  | *U. brizantha* | Brazil | MG | 2018 | Present study | U3 | SAMN19488867 |
| UbJA92 |  | *U. brizantha* | Brazil | MG | 2018 | Present study | U4 | SAMN19488868 |
| Ud8401 | Bd8401 | *Urochloa distachya* (Tropical signalgrass) | Philippines | Unknown | 1984 | Gladieux, et al. 2018 | U2 | SAMN08009543 |
| Um8309 | Bm8309 | *Urochloa mutica* (Buffalo grass) | Philippines | Los Baños, LB | 1983 | Borromeo, et al. 1993 | U1 | SAMN19488868 |
| Um88324 | Bm88324 | *U. mutica* | Philippines | Cabanatuan, NE | 1988 | Borromeo, et al. 1993 | U1 | SAMN08009544 |
| Um8946 | Bm8946 | *U. mutica* | Philippines | Imus, Cv | 1989 | Borromeo, et al. 1993 | U1 | SAMN19488870 |
| Up35 | Br35 | *Urochloa plantaginea* (Creeping signalgrass) | Brazil | PR | 1990 | Inoue, et al. 2017 | U3 | SAMN14144141 |
| GN0001 |  | *Zea mays (maize)* | Gabon | Wey |  | Pordel et al. 2020 | Ec | SAMEA7540994 |

^A^ Lineages/species are as follows: C1 = Cynodon1; C2 = Cynodon2; E1 = Eleusine1; E2 = Eleusine2; E3 = Eleusine3; Ec = Echinochloa; Er = Eragrostis; H = Hakonechloa; L1 = Lolium1; L2 = Lolium2; L3 = Lolium3; Lee = Leersia; Lu = Luziola; O = Oryza; P = Panicum; Pg = Pyricularia grisea; Pp = P. pennisetigena; Pu = P. urashimae; S = Setaria; St = Stenotaphrum; T - Triticum; U1 = Urochloa1; U2 = Urochloa2; U3 = Urochloa3; U4 = Urochloa4

**References for sequence datasets**

Borromeo, E. S., R. J. Nelson, J. M. Bonman and H. Leung, 1993 Genetic differentiation among isolates of *Pyricularia* infecting rice and weed hosts. Phytopathology 83**:** 393-399.

Castroagudín, V. L., A. L. D. Danelli, S. I. Moreira, J. T. A. Reges, G. de Carvalho, J. L. N. Maciel *et al.*, 2017 The wheat blast pathogen *Pyricularia graminis-tritici*; has complex origins and a disease cycle spanning multiple grass hosts. bioRxiv**:** 203455.

Castroagudín, V. L., S. I. Moreira, D. A. S. Pereira, S. S. Moreira, P. C. Brunner, J. L. N. Maciel *et al.*, 2016 *Pyricularia graminis-tritici*, a new *Pyricularia* species causing wheat blast. Persoonia 37**:** 199-216.

Chao, C.-C. T., and A. H. Ellingboe, 1991 Selection for mating competence in *Magnaporthe grisea* pathogenic to rice. Can. J. Bot. 69**:** 2130-2134.

Chiapello, H., L. Mallet, C. Guérin, G. Aguileta, J. Amselem, T. Kroj *et al.*, 2015 Deciphering Genome Content and Evolutionary Relationships of Isolates from the Fungus *Magnaporthe oryzae* Attacking Different Host Plants. Genome Biol. Evol. 7**:** 2896-2912.

Couch, B. C., I. Fudal, M.-H. Lebrun, D. Tharreau, B. Valent, P. van Kim *et al.*, 2005 Origins of host-specific populations of the blast pathogen *Magnaporthe oryzae* in crop domestication with subsequent expansion of pandemic clones on rice and weeds of rice. Genetics 170**:** 613-630.

Faivre-Rampant, O., J. Thomas, M. Allègre, J.-B. Morel, D. Tharreau, J.-L. Nottéghem *et al.*, 2008 Characterization of the model system rice–*Magnaporthe* for the study of nonhost resistance in cereals. New Phytol. 180**:** 899-910.

Farman, M., G. Peterson, L. Chen, J. Starnes, B. Valent, P. Bachi *et al.*, 2016 The *Lolium* Pathotype of *Magnaporthe oryzae* Recovered from a Single Blasted Wheat Plant in the United States. Plant Dis. 101**:** 684-692.

Gladieux, P., B. Condon, S. Ravel, D. Soanes, J. L. N. Maciel, A. Nhani *et al.*, 2018 Gene Flow between Divergent Cereal- and Grass-Specific Lineages of the Rice Blast Fungus *Magnaporthe oryzae*. mBio 9**:** e01219-01217.

Ikeda, K.-i., B. Van Vu, N. Kadotani, M. Tanaka, T. Murata, K. Shiina *et al.*, 2013 Is the fungus *Magnaporthe* losing DNA methylation? Genetics 195**:** 845-855.

Inoue, Y., T. T. P. Vy, K. Yoshida, H. Asano, C. Mitsuoka, S. Asuke *et al.*, 2017 Evolution of the wheat blast fungus through functional losses in a host specificity determinant. Science 357**:** 80.

Islam, M. T., D. Croll, P. Gladieux, D. M. Soanes, A. Persoons, P. Bhattacharjee *et al.*, 2016 Emergence of wheat blast in Bangladesh was caused by a South American lineage of *Magnaporthe oryzae*. BMC Biol. 14**:** 84.

Kumar, A., N. Sheoran, G. Prakash, A. Ghosh, S. K. Chikara, H. Rajashekara *et al.*, 2017 Genome Sequence of a Unique &lt;span class=&quot;named-content genus-species&quot; id=&quot;named-content-1&quot;&gt;Magnaporthe oryzae&lt;/span&gt; RMg-Dl Isolate from India That Causes Blast Disease in Diverse Cereal Crops, Obtained Using PacBio Single-Molecule and Illumina HiSeq2500 Sequencing. Genome Announc. 5**:** e01570-01516.

Pieck, M. L., A. Ruck, M. L. Farman, G. L. Peterson, J. P. Stack, B. Valent *et al.*, 2016 Genomics-Based Marker Discovery and Diagnostic Assay Development for Wheat Blast. Plant Dis. 101**:** 103-109.

Pordel, A., S. Ravel, F. Charriat, P. Gladieux, S. Cros-Arteil, J. Milazzo *et al.*, 2020 Tracing the Origin and Evolutionary History of *Pyricularia oryzae* Infecting Maize and Barnyard Grass. Phytopathology 111**:** 128-136.

Rahnama, M., T. D. Phillips and M. L. Farman, 2020 First Report of the Blast Pathogen, *Pyricularia oryzae*, on *Eragrostis tef* in the United States. Plant Dis. 104**:** 3266.

Shirke, M. D., H. B. Mahesh and M. Gowda, 2016 Genome-Wide Comparison of Magnaporthe Species Reveals a Host-Specific Pattern of Secretory Proteins and Transposable Elements. PLoS ONE 11**:** e0162458.

Soanes, D., L. S. Ryder, M. T. Islam and N. J. Talbot, 2017 Genome assemblies of Magnaporthe oryzae isolated from Bangladesh in 2016 and 2017. . figshare. Journal contribution.

Tosa, Y., K. Hirata, H. Tamba, S. Nakagawa, I. Chuma, C. Isobe *et al.*, 2004 Genetic Constitution and Pathogenicity of *Lolium* Isolates of *Magnaporthe oryzae* in Comparison with Host Species-Specific Pathotypes of the Blast Fungus. Phytopathology 94**:** 454-462.

Tosa, Y., W. Uddin, G. Viji, S. Kang and S. Mayama, 2007 Comparative Genetic Analysis of Magnaporthe oryzae Isolates Causing Gray Leaf Spot of Perennial Ryegrass Turf in the United States and Japan. Plant Dis. 91**:** 517-524.

Tredway, L. P., K. L. Stevenson and L. L. Burpee, 2003 Components of Resistance to *Magnaporthe grisea* in ‘Coyote’ and ‘Coronado’ Tall Fescue. Plant Dis. 87**:** 906-912.

Urashima, A. S., Y. Hashimoto, L. D. Don, M. Kusaba, Y. Tosa, H. Nakayashiki *et al.*, 1999 Molecular Analysis of the Wheat Blast Population in Brazil with a Homolog of Retrotransposon MGR583. Jap. J. Phytopathol. 65**:** 429-436.

Xue, M., J. Yang, Z. Li, S. Hu, N. Yao, R. A. Dean *et al.*, 2012 Comparative Analysis of the Genomes of Two Field Isolates of the Rice Blast Fungus *Magnaporthe oryzae*. PLoS Genet. 8**:** e1002869.

Yasuhara-Bell, J., K. F. Pedley, M. Farman, B. Valent and J. P. Stack, 2018 Specific Detection of the Wheat Blast Pathogen (*Magnaporthe oryzae Triticum*) by Loop-Mediated Isothermal Amplification. Plant Dis. 102**:** 2550-2559.

Yoshida, K., D. G. O. Saunders, C. Mitsuoka, S. Natsume, S. Kosugi, H. Saitoh *et al.*, 2016 Host specialization of the blast fungus *Magnaporthe oryzae* is associated with dynamic gain and loss of genes linked to transposable elements. BMC Genomics 17**:** 370.

Zhong, Z., J. Norvienyeku, M. Chen, J. Bao, L. Lin, L. Chen *et al.*, 2016 Directional Selection from Host Plants Is a Major Force Driving Host Specificity in *Magnaporthe* Species. Scientific Reports 6**:** 25591.

**Supplementary Table 2.** Comparative genomic analysis of MoTeR relic loci. MoTeR relics were identified in MinION assemblies of each genome and orthologous MoTeR copies were identified based on chromosome synteny and identical flanking sequences.

| **Chr** | **Strain** | **also present in:** | **3’ end Position** | **Relic length (bp)** | **3’ end sequence^A^** |
| --- | --- | --- | --- | --- | --- |
| Chr1 | CD156 | - | 215205 | 40 | CGCGAATTAAAA**CCCTAACCCTTA** |
| Chr1 | CD156 | - | 753727 | 4,277 | GCGCGAATTAAGA**CCCAT** |
| Chr1 | CD156 | - | 5749080 | 40 | CGCGAATTAAAA**CCCTA**TA |
| Chr1 | B71 | - | 6127382 | 40 | CGCGAATTAAAA**CCCTA** |
| Chr1 | U233 | - | 4947590 | 2,089 | CGCGAATTAAAA**CCCTAA** |
| Chr1 | Arcadia | - | 5692246 | 26 | CGCGAATTAAAA**CCCT** |
| Chr1 | Arcadia | - | 5425062 | 97 | CGCGAATTAAAA**CCCTAACCCT** |
| Chr1 | Arcadia | - | 5374231 | 140 | **TTAGGGTTAGGG**TTTTAATTCGCA |
| Chr2 | LpKY97 | - | 25940 | 98 | ACGCGAATTAAAA**CCCTAA** |
| Chr2 | LpKY97 | FH, B71, Arcadia | 28007 | 117 | ^B^ AAAATTAAGCGC |
| Chr2 | LpKY97 | Guy11, Bm88324 | 42376 | 40 | GCGCGAATTAAAA**CCCTAACCCTAAC** |
| Chr2 | FH | LpKY97, B71, Arcadia | 645364 | 109 | **GGTTAGGG**TTTTAATTCGCG |
| Chr2 | CD156 | - | 7550702 | 31 | CGCGAATTAAAA**CCCTAAC** |
| Chr2 | CD156 | - | 7553167 | 40 | CGCGAATTAAAA**CCCTA** |
| Chr2 | B71 | LpKY97, FH, Arcadia | 39554 | 118 | **GGTTAGGG**TTTTAATTCGCG |
| Chr2 | Guy11 | LpKY97, Bm88324 | 44503 | 40 | CGCGAATTAAAA**CCCTAACC**GTAA |
| Chr2 | Guy11 | Bm88324 | 45642 | 102 | CGCGAAATAAAA**CCC**AAA |
| Chr2 | Guy11 | - | 5458604 | 40 | CGCGAATTAAAA**CCCTAA** |
| Chr2 | Arcadia | LpKY97, FH, B71 | 11850 | 118 | **GGTTAGGG**TTTTAATTCGCG |
| Chr2 | Bm88324 | LpKY97, Guy11 | 3176 | 39 | CGCGAATTAAAA**CCCTAACCCTAA** |
| Chr2 | Bm88324 | Guy11 | 4311 | 98 | CGCGAAATAAAG**CCC**AAAA |
| Chr3 | LpKY97 | FH, Arcadia, Bm88324 | 152722 | 40 | **GGGTTAGGG**TTTTAATTCGCGT |
| Chr3 | LpKY97 | FH, CD156, B71 | 2548296 | 41 | **AGGGTTAGGG**TTTTAATTCGCGT |
| Chr3 | LpKY97 | FH, CD156, B71 | 2742283 | 40 | ACGCGAATTAAAA**CCCTAACCCTA** |
| Chr3 | LpKY97 | FH, CD156, B71 | 2751680 | 41 | **GTTAGG**TTTTAATTCGCG |
| Chr3 | LpKY97 | FH, CD156, B71 | 2787030 | 31 | **GGTTGGGG**TTTTAATTCGCG |
| Chr3 | LPKY97 | FH, *CD156 | 7355697 | 114 | **TTAGGG**TTTTAATTCGCGC |
| Chr3 | FH | LpKY97, Arcadia, Bm88324 | 169997 | 40 | **GGGTTAGGG**TTTTAATTCGCG |
| Chr3 | FH | LpKY97, CD156, B71 | 2553536 | 41 | **GGGTTAGGG**TTTTAATTCGCG |
| Chr3 | FH | LpKY97, CD156, B71 | 2749138 | 40 | CGCGAATTAAAA**CCCTAACCCTA** |
| Chr3 | FH | LpKY97, CD156, B71 | 2758545 | 41 | **GTTAGGG**TTTTAATTCGCG |
| Chr3 | FH | LpKY97, CD156, B71 | 2793917 | 40 | **GGTTGGGG**TTTTAATTCGCG |
| Chr3 | FH | LpKY97, CD156, B71 | 7315089 | 119 | **TTAGGG**TTTTAATTCGCG |
| Chr3 | CD156 | LpKY97, FH, B71 | 2424482 | 40 | **AGGGTTAGGG**TTTTAATTCGCG |
| Chr3 | CD156 | LpKY97, FH, B71 | 2621949 | 40 | CGCGAATTAAAA**CCCTAACCCTA** |
| Chr3 | CD156 | LpKY97, FH, B71 | 2625549 | 41 | **GTTAGGG**TTTTAATTCGCG |
| Chr3 | CD156 | LpKY97, FH, B71 | 2660716 | 31 | **GGTTGGGG**TTTTAATTCGCG |
| Chr3 | CD156 | LpKY97, FH | 7124927 | 15 | **TTAGGG**TTTAATTCGCT |
| Chr3 | B71 | LpKY97, FH, CD156 | 2523720 | 41 | **GTCAGGGTTAGGG**TTTTAATTCGCG |
| Chr3 | B71 | LpKY97, FH, CD156 | 2719208 | 40 | CGCGAATTAAAA**CCCTAACCCTA** |
| Chr3 | B71 | LpKY97, FH, CD156 | 2722828 | 41 | **GTTAGGG**TTTTAATTCGCG |
| Chr3 | B71 | LpKY97, FH, CD156 | 2758440 | 31 | **GGTTGGGG**TTTTAATTCGCG |
| Chr3 | B71 | - | 7468533 | 40 | **TAGGGTTAGGG**TTTTAATTCGCG |
| Chr3 | Arcadia | LpKY97, FH, Bm88324 | 165684 | 40 | **GGGTTAGGG**TTTTAATTCGCG |
| Chr3 | Bm88324 | LpKY97, FH, Arcadia | 148531 | 40 | **GGGTTAGGG**TTTTAATTCGCG |
| Chr4 | Arcadia | - | 15722 | 239 | CGCGAATTAAAA**CCCTAACCCTAA** |
| Chr4 | Bm88324 | - | 5007462 | 131 | CGCGAATTAAAA**CCCTAA** |
| Chr5 | LpKY97 | FH | 4395439 | 147 | CGCGAATTAAAA**CCCTAACCC** |
| Chr5 | FH | LpKY97 | 4423681 | 149 | CGCGAATTAAAA**CCCTAACCC** |
| Chr5 | Guy11 | - | 185204 | 140 | **TTATGGTGAGGG**TTTTAATTCGCG |
| Chr5 | Arcadia | - | 122817 | 105 | CGCGAGTTAGAA**CCCTAACCCTAA** |
| Chr5 | Arcadia | - | 4700151 | 166 | **AGGGTTAGGG**TTTTAATTCGCG |
| Chr6 | LpKY97 | FH, CD156, B71 | 83149 | 31 | CGCGAATTAAAA**CCCTAA** |
| Chr6 | LpKY97 | FH, CD156, B71 | 2132281 | 40 | **GTTGGGG**TTTTAATTCGCG |
| Chr6 | LpKY97 | FH, CD156, B71 | 3271965 | 131 | CGCGAATTAAAA**CCTAACCATCCCA** |
| Chr6 | FH | LpKY97, CD156, B71 | 83149 | 31 | CGCGAATTAAAA**CCCTAA** |
| Chr6 | FH | LpKY97, CD156, B71 | 2144774 | 39 | **TTAGGG**TTTTAATTCGCG |
| Chr6 | FH | LpKY97, CD156, B71 | 3282558 | 131 | CGCGAATTAAAA**CCCTAA** |
| Chr6 | CD156 | LpKY97, FH, B71 | 95554 | 31 | CGCGAATTAAAA**CCCTAA** |
| Chr6 | CD156 | LpKY97, FH, B71 | 2153119 | 40 | **TTGGGG**TTTTAATTCGCG |
| Chr6 | CD156 | LpKY97, FH, B71 | 3282214 | 132 | CGCGAATTAAAA**CCCTAACC** |
| Chr6 | B71 | LpKY97, FH, CD156 | 78239 | 31 | CGCGAATTAAAA**CCCTAA** |
| Chr6 | B71 | LpKY97, FH, CD156 | 2138379 | 40 | **GTTGGGG**TTTTAATTCGCG |
| Chr6 | B71 | LpKY97, FH, CD156 | 3275257 | 135 | CGCGAATTAAAA**CCCTAACC** |
| Chr6 | Arcadia | - | 22696 | 1146 | CGCGAATTAAAA**CCCTAACCCTA** |
| Chr7 | LpKY97 | FH, CD156, Bm88324, B71 | 3688512 | 38 | CGCGAATTAAAA**CCTTAACCCTAA** |
| Chr7 | LpKY97 | FH, CD156 | 3825400 | 63 | **TTAGGTGTGGG**TTTTAAATCGCG |
| Chr7 | LpKY97 | FH, CD156 | 3835928 | 110 | CGCGAATTGAAA**CCCTAACCCTAA** |
| Chr7 | FH | LpKY97, CD156, Bm88324, B71 | 3694340 | 37 | CGCGAATTAAAA**CCTTAACCCTAA** |
| Chr7 | FH | LpKY97, CD156 | 3836980 | 63 | **TTAGGGTTAGGG**TTTTAAATCGCG |
| Chr7 | FH | LpKY97, CD156 | 3841696 | 116 | CGCGAATTGAAA**CCCTAACCCTAA** |
| Chr7 | CD156 | LpKY97, FH, Bm88324, B71 | 3761013 | 38 | CGCGAATTAAAA**CCTTAACCCTAA** |
| Chr7 | CD156 | LpKY97, FH | 3893775 | 63 | **TTAGGGTGTGGG**TTTTAAATCGCG |
| Chr7 | CD156 | LpKY97, FH | 3896604 | 105 | CGCGAATTGAAA**CCCTAACCCTAA** |
| Chr7 | B71 | Bm88324 | 3755038 | 59 | **TAAGGGTTAGGG**TTTTAAATTCGCG |
| Chr7 | B71 | LpKY97, FH, Bm88324, CD156 | 3755790 | 59 | CGCGAATTAAAA**CCTTAACCCTAA** |
| Chr7 | Arcadia | - | 2960437 | 132 | CGCGAATTAAAA**CCCTAA** |
| Chr7 | Bm88324 | - | 560250 | 94 | **AGGGTTAGGG**TTTTAATTCGCG |
| Chr7 | Bm88324 | B71 | 4570424 | 112 | **GTTAAGGTTAGG**TTTTAAATTCGCG |
| Chr7 | Bm88324 | B71, LpKY97, FH,, CD156 | 4571156 | 124 | CGCGAATTAAAA**CCTTAAACCCAAA** |

**^A^** MoTeR 3' terminus sequence is underlined. Flanking telomere vestige is highlighted in bold.

**^B^** No obvious telomere sequence detected.

**Supplementary Table 3.** MoTeR relic positions and their associated duplications in the CD156 genome. The positions of the sequences adjacent to the relic are listed first, then the duplicated copy. Relics without duplications are also listed.

| **Chr** | **Relic 3’ position** | | **Duplication type** | | | **Start (5’)** | | **End**  **(3’)** | | **Length** | | **Dupl. Chr** | **Dupl. start** | **Dupl. end** |
| --- | --- | --- | --- | --- | --- | --- | --- | --- | --- | --- | --- | --- | --- | --- |
| Chr1 | 215205 | | 3’ | | | 215231 | | 216153 | | 922 | | Chr1 | 82487 | 83397 |
| Chr1 | 753727 | | - | | | - | | - | | - | | - | - | - |
| Chr1 | 5749080 | | 3’ | | | 5749041 | | 5749080 | | 242 | | Chr3 | 2488147 | 2488390 |
| Chr1 | 5749080 | | Relic + 5’ | | | 5748567 | | 5749085 | | 518 | | Chr6 | 95111 | 95570 |
| Chr2 | 7550702 | | Relic + 5’ | | | 7550142 | | 7550719 | | 577 | | Chr6 | 94948 | 95571 |
| Chr2 | 7553167 | | - | | | - | | - | | - | | - | - | - |
| Chr3 | 2424482 | | 3’ | | | 2423615 | | 2424461 | | 849 | | Chr5 | 4504267 | 4505293 |
| Chr3 | 2621949 | | 3’ | | | 2621959 | | 2622189 | | 230 | | Chr1 | 1600219 | 1600452 |
| Chr3 | 2625538 | | 3’ | | | 2622713 | | 2625525 | | 2812 | | Chr5 | 191310 | 204348 |
| Chr3 | 2660705 | | 3’ | | | 2658586 | | 2660695 | | 2230 | | Chr5 | 213434 | 215664 |
| Chr6 | 95565 | | Relic + 5’ | | | 94948 | | 95571 | | 623 | | Chr2 | 7550142 | 7550719 |
| Chr6 | | 2153119 | | - | - | | - | | - | | - | | - | - |
| Chr6 | | 3282214 | | - | - | | - | | - | | - | | - | - |
| Chr7 | | 3761013 | | - | - | | - | | - | | - | | - | - |
| Chr7 | | 3896608 | | - | - | | - | | - | | - | | - | - |
